# Cryptotanshinone inhibits ovarian tumor growth and metastasis by degrading c-Myc and attenuating the FAK signaling pathway

**DOI:** 10.3389/fcell.2022.959518

**Published:** 2022-09-28

**Authors:** Huijun Guo, Wenjing Zhang, Jiaxing Wang, Guannan Zhao, Yaohong Wang, Bing-Mei Zhu, Peixin Dong, Hidemichi Watari, Baojin Wang, Wei Li, Gabor Tigyi, Junming Yue

**Affiliations:** ^1^ Department of Pathogen Biology and Immunology, College of Life Science, Jiangxi University of Traditional Chinese Medicine, Nanchang, China; ^2^ Department of Pathology and Laboratory Medicine, College of Medicine, The University of Tennessee Health Science Center, Memphis, TN, United States; ^3^ Department of Genetics, Genomics and Informatics, College of Medicine, The University of Tennessee Health Science Center, Memphis, TN, United States; ^4^ Department of Pharmaceutical Sciences, College of Pharmacy, The University of Tennessee Health Science Center, Memphis, TN, United States; ^5^ Department of Pathology, Immunology and Microbiology, Vanderbilt University, Nashville, TN, United States; ^6^ Regenerative Medicine Research Center, West China Hospital, Sichuan University, Chengdu, China; ^7^ Department of Obstetrics and Gynecology, Hokkaido University Graduate School of Medicine, Sapporo, Japan; ^8^ Department of Gynecology and Obstetrics, Third Affiliated Hospital, Zhengzhou University, Zhengzhou, China; ^9^ Department of Physiology, College of Medicine, The University of Tennessee Health Science Center, Memphis, TN, United States

**Keywords:** cryptotanshinone, ovarian cancer, c-Myc, FAK, ubiquitination, metastasis

## Abstract

Cryptotanshinone (CT), a natural compound derived from *Salvia miltiorrhiza* Bunge that is also known as the traditional Chinese medicine Danshen, exhibits antitumor activity in various cancers. However, it remains unclear whether CT has a potential therapeutic benefit against ovarian cancers. The aim of this study was to test the efficacy of CT in ovarian cancer cells *in vitro* and using a xenograft model in NSG mice orthotopically implanted with HEY A8 human ovarian cancer cells and to explore the molecular mechanism(s) underlying CT’s antitumor effects. We found that CT inhibited the proliferation, migration, and invasion of OVCAR3 and HEY A8 cells, while sensitizing the cell responses to the chemotherapy drugs paclitaxel and cisplatin. CT also suppressed ovarian tumor growth and metastasis in immunocompromised mice orthotopically inoculated with HEY A8 cells. Mechanistically, CT degraded the protein encoded by the oncogene c-Myc by promoting its ubiquitination and disrupting the interaction with its partner protein Max. CT also attenuated signaling via the nuclear focal adhesion kinase (FAK) pathway and degraded FAK protein in both cell lines. Knockdown of c-Myc using lentiviral CRISPR/Cas9 nickase resulted in reduction of FAK expression, which phenocopies the effects of CT and the c-Myc/Max inhibitor 10058-F4. Taken together, our studies demonstrate that CT inhibits primary ovarian tumor growth and metastasis by degrading c-Myc and FAK and attenuating the FAK signaling pathway.

## Introduction

Ovarian cancer has the highest mortality rate among gynecological malignances yet has only limited options for therapeutic intervention (Nash and Menon, 2020). Currently the major strategy for treating ovarian cancer patients is to perform debulk surgery in combination with chemotherapy, targeted therapy, or hormone therapy. However, ovarian cancer frequently recurs due to metastasis and resistance to chemotherapy drugs (Martins et al., 2014; Dilruba and Kalayda, 2016; Lazarevic et al., 2017; Kan et al., 2018; Ma et al., 2020). Therefore, it is essential to develop new chemotherapy drugs with reduced cytotoxicity and improved efficacy to overcome the chemoresistance. Natural products are an alternative resource for their antitumor activity, ability to overcome multidrug resistance due to multiple potential gene targets, and lower toxicity than conventional chemotherapy drugs (El-Senduny et al., 2016; de Oliveira Junior et al., 2018). At present, over 60% of approved cancer drugs are derived from natural plants (Ijaz et al., 2018).

Cryptotanshinone (CT) is a natural compound extracted from the root of *Salvia miltiorrhiza* Bunge (Red Sage) that inhibits cell proliferation, motility/invasion, and angiogenesis by targeting STAT3 in several cancers (Ge et al., 2015; Yan et al., 2016; Chen et al., 2017; Ke et al., 2017; Dong et al., 2018; Yang et al., 2018; Qin et al., 2019). It remains unknown whether CT exhibits antitumor activity in ovarian cancer *in vivo*, although it has been reported to have inhibitory effects on ovarian tumor cell growth *in vitro* (Jiang et al., 2017; Yang et al., 2018). The molecular mechanism underlying CT antitumor activity remains completely unknown.

The transcription factor c-Myc is upregulated or amplified in most cancer types, including ovarian cancer (Jing et al., 2016). The *c-Myc* oncogene is located in the same genomic locus (8q24) as the oncogene *FAK*, in a region known to be associated with aggressive cancer phenotypes, recurrence, and metastasis (Ehlers et al., 2005; Zhao et al., 2018; Vasmatzis et al., 2019). Both *c-Myc* and *FAK* are upregulated in over 40% cases of high grade serous carcinoma (HGSCs) and amplified in 65% of HGSCs, respectively, whereas p53 was mutated in over 90% of patients (Zeng et al., 2018). Previous studies showed that Myc transcriptionally activates FAK expression by binding to the promoter of FAK (Beierle et al., 2007). Therefore, targeting c-Myc and FAK represents a potential novel strategy for the development of new ovarian cancer therapies. However, the ubiquitous expression and cell nuclear localization of c-Myc make it very difficult to directly target c-Myc using small molecule inhibitors. Several c-Myc inhibitors, including JQ1, 10058-F4, 10074-G5, and Omomyc, were developed for cancer therapy on the basis of their targeting of c-*Myc* transcription or the interaction between c-Myc and its partner protein Max, thereby blocking their heterodimerization and subsequent binding to the E-box of the *Myc* promoter, leading to inactivation of downstream target gene expression (Wang et al., 2014; Allen-Petersen and Sears, 2019).

The FAK signaling pathway is activated in several different cancers and multiple small molecule inhibitors of FAK have been developed, although only four small molecule inhibitors of FAK, including GSK2256098, VS-4078, VS-6062, and VS-6063 (defactinib), have been used in clinical trials for cancer therapy Sulzmaier et al. (2014), McLean et al. (2003), Mohanty et al. (2020). However, the most advanced compound, VS-6063, failed in clinical trials to treat malignant pleural mesothelioma Cromm et al. (2018). FAK exerts its regulatory activity in both kinase-dependent and kinase-independent pathways. However, these inhibitors only block FAK’s kinase activity and fail to degrade FAK so that FAK retains its kinase-independent function as a scaffold protein. Consequently, a new generation drug PROTAC (Proteolysis Targeting Chimeric Molecule) was developed to inhibit both FAK’s kinase activity and its scaffold protein function. PROTAC showed greater efficacy than its parent, FAK inhibitor VS6063, in inhibiting tumor cell invasion Cromm et al., (2018), Huo et al., (2022).

Targeting both c-Myc and FAK is an important strategy for ovarian cancer therapy because both genes are highly amplified and upregulated in ovarian cancer cells. In this study, we assessed the antitumor effects of CT in ovarian cancer cells *in vitro* and tested its efficacy using orthotopic ovarian cancer mouse models *in vivo*. We uncovered a novel molecular mechanism underlying the antitumor properties of CT, characterized by its degradation of c-Myc and FAK.

## Materials and methods

### Cell culture

Two different ovarian cancer cell lines, OVCAR3 and HEY A8, were chosen for this study due to their different p53 status, since p53 is mutated in OVCAR3 cells and wildtype (WT) in HEY A8 cells. Both cell lines were purchased from the National Cancer Institute (Bethesda, MD) and cultured in RPMI 1640 medium supplemented with 10% fetal bovine serum (FBS, Hyclone; Logan, UT), 100 U/ml penicillin, and 100 μg/ml streptomycin (Invitrogen; Carlsbad, CA).

### MTT cell proliferation assay

The MTT cell proliferation assay kit was purchased from ATCC and used to measure cell proliferation and viability. OVCAR3 or HEY A8 cells were seeded to 96-well plates at 3,000 cells/well density and incubated overnight. The next day, cells were treated with 0.1% DMSO or different doses of CT and cultured for 24 or 48 h. The MTT reagent (10 μl) was added to each well and incubated for 4 h. The reaction was stopped by adding 150 μl DMSO and the plates were incubated at room temperature (RT) in the dark for 10 min. Cell proliferation was assessed by measuring the absorbance at 570 nm. Cell viability was also measured with acridine orange/propidium solutions and live (green) and dead (red) cells were counted using a Luna-FL automatic cell counter.

### Colony formation assay

Three hundred ovarian cancer cells per well were plated and cultured for 72 h before treatment. Subsequently, the cells were treated with DMSO or 0.5 μM CT in triplicate for 14 days. On day 14, the colonies were fixed, stained with crystal violet (Sigma, St. Louis, MO, USA) dissolved in 10% ethanol, and the number of colonies was counted.

### Cell migration and invasion assay

Briefly, OVCAR3 or HEY A8 cells were treated with 20 µM CT in 0.1% DMSO or with 0.1% DMSO vehicle control for 24 h. Modified Transwell chambers (BD Falcon™, San Jose, CA) were inserted into 24-well cell culture plates. RPMI1640 medium containing 10% FBS served as the chemoattractant in the lower chamber. Then 3×10^5^ cells in 300 μl serum-free RPMI1640 were added to the upper chamber, which was left uncoated or coated with Matrigel as described previously for migration and invasion assays, respectively (El-Senduny et al., 2016). After incubation for 24 h, the non-migrated cells in the upper chamber were removed gently with a cotton swab, while the migrated or invaded cells located on the surface of the lower chamber were stained with crystal violet (CV) or hematoxylin-eosin (HE) and counted.

### Immunofluorescence assay

Following exposure to 0.1% DMSO, 10 μM CT, or CT combined with chemotherapy drugs for 24 h, the cells were washed with PBS, fixed in 99.8% methanol for 20 min and permeabilized with 0.1% Triton X-100 in PBS containing 0.5% BSA (Sigma-Aldrich) for 1 h. To detect the expression of cleaved caspase-3 in cells, the cells were fixed in 4% (W/V) paraformaldehyde (PFA) and incubated with blocking buffer containing 5% normal goat serum in PBS for 1 h. The cells were subsequently incubated with antibodies specific for cleaved caspase-3, c-Myc, and ubiquitin (Cell Signaling, diluted 1:200 in blocking buffer) at 4°C overnight. After washing with PBS, cells were incubated with Alexa Fluor 488- or Alexa Fluor 568-conjugated secondary antibodies (A27023; 1:1,000 Thermo Fisher Scientific, Inc.) at RT in the dark for 45 min. The cells were washed with PBS, incubated in the 100 ng/ml DAPI solution, and imaged with a fluorescence microscope.

### Generation of c-Myc knockdown ovarian cancer cells using lentiviral CRISPR/Cas9 nickase vector

The lentiviral CRISPR/Cas9 nickase-mediated c-Myc gene editing vectors were constructed by synthesizing and annealing two gRNAs (5′TTG​GTG​AAG​CTA​ACG​TTG​AG and 5′CTA​TGA​CCT​CGA​CTA​CGA​CT) and subcloning them in the BsmII site of the lentiviral Lentiguide-puro vector (#52963, Addgene, Watertown, MA). CRISPR/Cas9 nickase expression was driven by the EF1a promoter in the LentiCas9-blast vector (catalog #52962, Addgene). Lentiviruses were produced by packaging the vectors in 293FT cells as described previously (Yue et al., 2010). Stable c-Myc KD cell lines were established by transducing the OVCAR3 or HEY A8 cells with the LentiCas9-blast Cas9 nickase vector, selecting positive cells in 10 μg/ml blasticidin, and subsequently transducing these cells with the lentiviral CRISPR/Cas9 nickase-mediated c-Myc gRNA vector and selecting positive cells with 1 μg/ml puromycin. LentiCas9-blast without gRNAs was used as the control vector.

### Immunoblot

Immunoblot analysis was performed as previously described (Zhao et al., 2017). In the MG132 proteasome inhibitor experiments, cells were treated with 10 μM CT and 5 µM MG132 (Medexpress, Monmouth Junction, NJ) for 24 h and collected for immunoblot analysis. In the cycloheximide (CHX, Medexpress) pulse-chase assay, cells were treated with 10 μM CT for 24 h, when 50 μg/ml of CHX was added, and the cells were collected at different time points thereafter. Primary antibodies used for immunoblot analysis included cleaved-PARP, cleaved-caspases-3, c-Myc, Max, FAK, p-FAK (1:1,000, Cell Signaling), Ubiquitin, and GAPDH (1:1,000, Santa Cruz), secondary antibodies were HRP conjugated anti-rabbit or anti-mouse (1:10000, Santa Cruz), and the identified bands were analyzed by Image J.

### Co-immunoprecipitation assay

To detect the physical interaction between c-Myc and Max and ubiquitinylated c-Myc species, OVCAR3 cells were treated with 10 μM CT for 24 h. To prevent CT induced c-Myc degradation, OVCAR3 cells were treated with 10 µM CT and 5 µM MG132 for 6 h. In each case, cells were lysed with 25 mM Tris, 150 mM NaCl, 1 mM EDTA, 1% NP-40 and 5% glycerol buffer (pH 7.4). The cell lysates were pre-cleared with Control Agarose Resin from the Pierce Classic IP Kit (Thermo, 26146) at 4°C for 1 h. The protein concentration in the lysates was determined by BCA™ protein assay kit (Pierce) and a volume of lysate containing 1.5 mg of protein was incubated with either Max antibodies (Cell Signaling) or c-Myc antibodies (Santa Cruz, N-262) at 4°C overnight. Immune complexes were captured by incubation with Pierce Protein A/G Agarose for 2 h, washed three times with lysis buffer and once with conditioning buffer, and eluted with the elution buffer included in the kit. The eluted fractions were analyzed by immunoblot using c-Myc or ubiquitin (Santa Cruz, SC-8017) antibodies, respectively.

### Orthotopic ovarian cancer mouse model

All animal experiments were performed in accordance with a protocol approved by the University of Tennessee Health Science Center (UTHSC) Institutional Animal Care and Use Committee (IACUC). Two-month-old immunocompromised NOD Cg-Prkdcscid Il2rgtm1Wjl/SzJ (NSG) female mice were purchased from the Jackson Laboratory. HEY A8 cells were labeled with luciferase by using the lentiviral vector pEF1a-Luc2. 5 × 10^5^ cells were injected intrabursally (i.b.) into the NSG females under dissecting microscopy. After 1 week, the mice were randomized into groups of five and treated with vehicle (10% DMSO +90% corn oil) or with CT (10 mg/kg bodyweight) via intraperitoneal (i.p.) injection 5 days per week for 3 weeks. Primary ovarian tumor growth and metastatic dissemination in NSG mice were monitored weekly using a live animal imaging system (PerkinElmer) by measuring bioluminescence after i. p. Injection of 0.25 ml D-luciferin (15 mg/ml). All mice were sacrificed after 3 weeks. Tumors were harvested for histology, immunofluorescent staining, and immunoblot to determine c-Myc and FAK expressions.

### Computational dock modeling

Maestro software (Schrödinger) was used for molecular modeling. Structure of Myc-Max-DNA was sourced from PDB (1NKP) and preprocessed through Protein Preparation wizard. Possible binding sites were generated via SiteMap wizard. Structure of CT was input through 2D sketcher and prepared with LigPrep wizard. Receptor grid was generated based on the SiteMap output Sites 1–5. Docking results and docking scores were obtained through Ligand Docking wizard.

### Statistical analysis

Significant differences were determined from at least three independent experiments performed in triplicate by Student’s t*-*test, and data were presented as mean values ±SD; differences with *p* < 0.05 was considered statistically significant.

## Results

### CT inhibits ovarian cancer cell proliferation and survival

**FIGURE 1:**
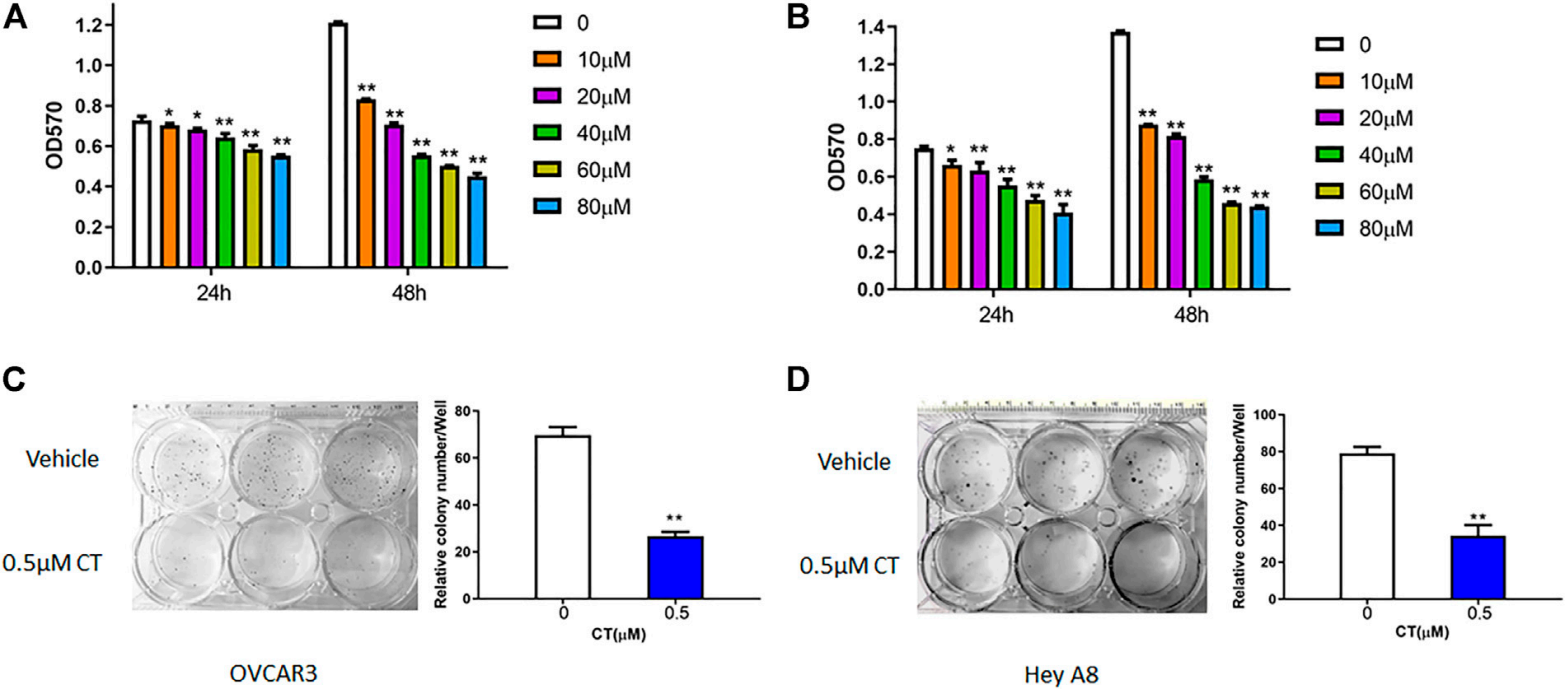
CT inhibits ovarian cancer cell proliferation. **(A,B)** MTT proliferation assay in OVCAR3 **(A)** and HEY A8 **(B)** cells treated with different doses of CT for 24 or 48 h. **(C,D)** Colony formation assay of OVCAR3 and HEY A8 cells treated with 0.5 μM CT for 14 days. Representative images and average colony numbers are shown. Data were presented as mean values ±SD. *p < 0.05, **p < 0.01, versus control group (*n* = 3).

To assess whether CT treatment affects ovarian cancer cell growth, we measured cell proliferation and viability in OVCAR3 and HEY A8 cells treated with different concentrations of CT for 24 or 48 h. In both cell lines, CT inhibited the proliferation in a dose- and time- dependent manner (Figures 1A,B). CT significantly inhibited cell proliferation and viability from 10 to 80 µM after 24 h or 48 h treatment. We also examined cell viability using acridine orange/propidium staining at the low doses of 10 and 20 µM and found that CT did not induce significant cytotoxicity (Supplementary Figures S1A,B). We further assessed how CT affects clonogenic survival using a colony formation assay, in which both cell lines were treated with 0.5 μM CT for 14 days. CT significantly inhibited colony growth (Figures 1C,D).

### CT suppresses ovarian cancer cell migration and invasion

**FIGURE 2:**
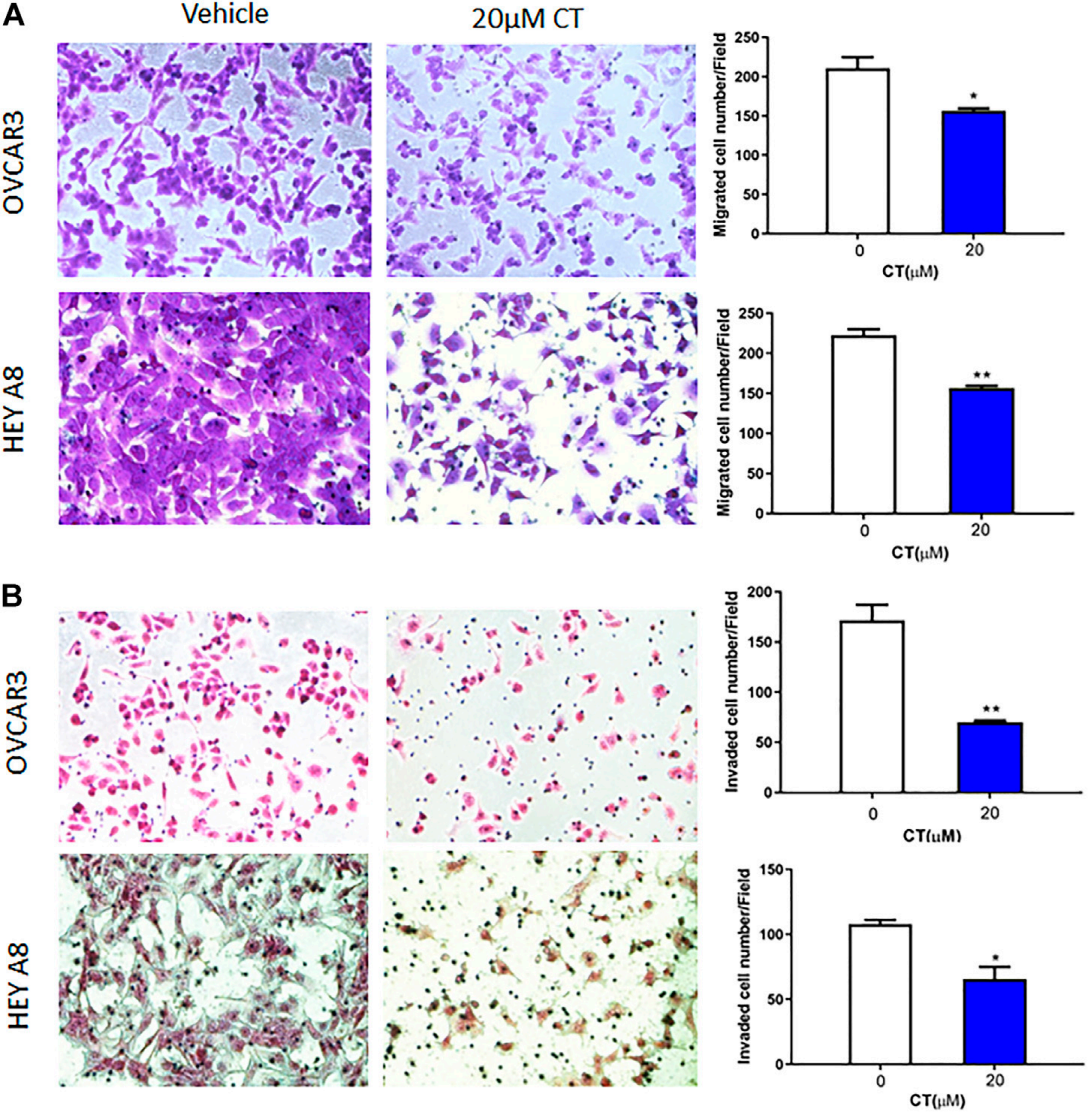
CT inhibits ovarian cell migration and invasion. **(A)** Transwell migration assay was performed in OVCAR3 and HEY A8 cells treated with 20 μM CT for 24 h. Migrated cells were stained using crystal violet and counted. **(B)** Matrigel-coated transwell assay was performed in OVCAR3 and HEY A8 cells treated with 20 μM CT for 24 h. Invaded cells were stained using HE and counted. Representative images are shown. Quantification of migration and invasion was presented as mean values ±SD from three independent experiments. *p < 0.05, **p < 0.01.

Both migration and invasion contribute to ovarian tumor metastasis. To examine whether CT regulates ovarian cancer cell migration and invasion, we treated OVCAR3 and HEY A8 cells with 20 μM CT for 24 h. Cell migration was determined in transwell plates, whereas invasion was examined using Matrigel coated transwell inserts. CT significantly inhibited migration and invasion in both OVCAR3 and HEY A8 cells (Figures 2A,B).

### CT inhibits c-Myc and FAK expression comparable to that of c-Myc inhibitor 10058-F4

**FIGURE 3:**
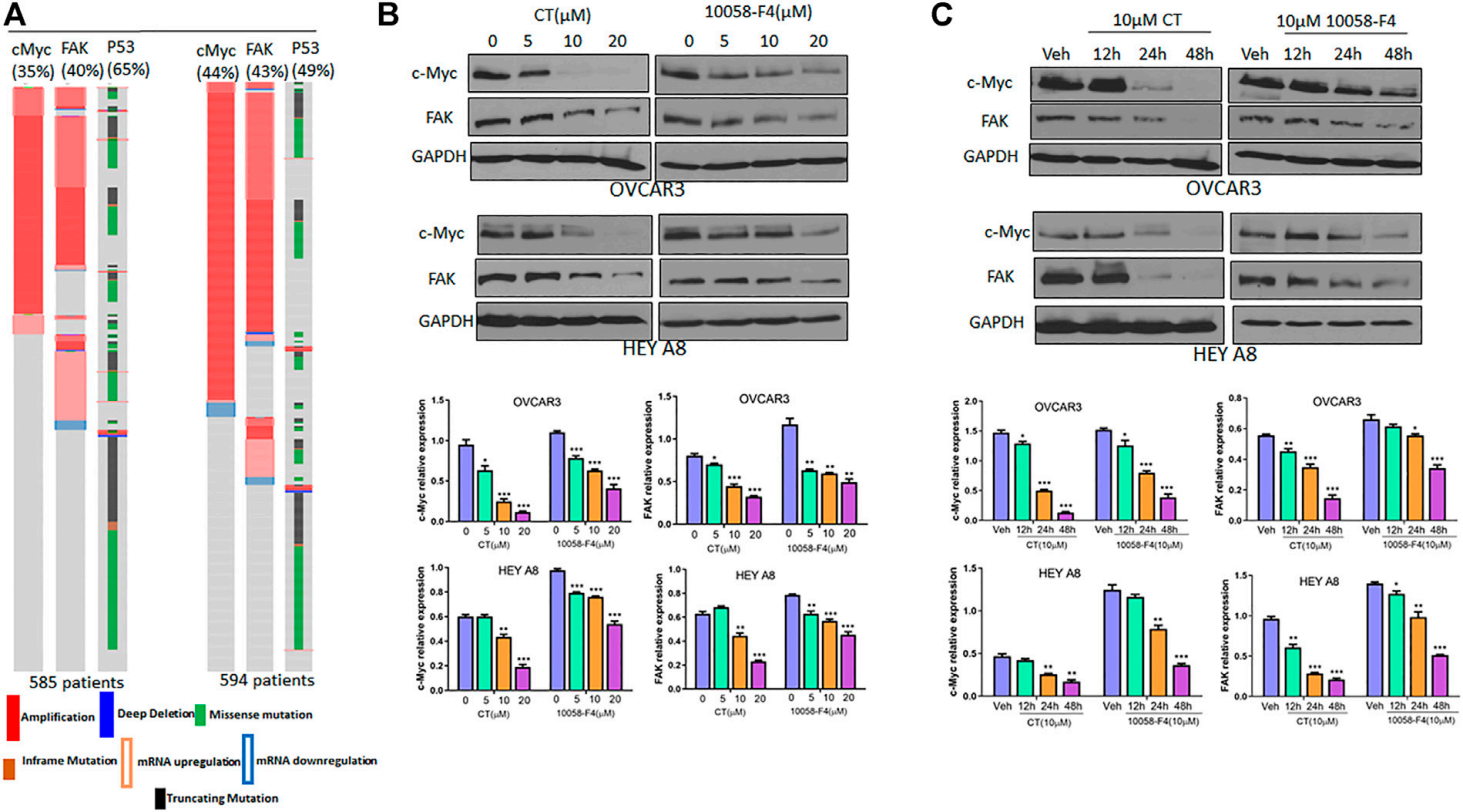
CT inhibits c-Myc and FAK expression in ovarian cancer cells. **(A)** *c-Myc* and *FAK* genes were amplified or upregulated in HGSC in two different datasets. The alteration for each gene was indicated as percentage among those patients. **(B)** Immunoblot and densitometry analysis of c-Myc and FAK expression following treatment of OVCAR3 and HEY A8 cells for 24 h with different doses of CT or 10058-F4. **(C)** Immunoblot and densitometry analysis of c-Myc and FAK expression in OVCAR3 and HEY A8 cells following treatment with 10 μM CT or 10058-F4 at different time points. Data are presented as mean values ±SD. *p < 0.05, **p < 0.01, ***p < 0.001.

We examined *c-Myc* and *FAK* genetic alterations in patients with high-grade serous carcinoma (HGSC) that had been previously deposited in The Cancer Genome Atlas (TCGA) database. The *c-Myc* and *FAK* genes were amplified or upregulated in 35 and 40%, respectively, of 585 patients in the TCGA Pan Cancer Atlas, whereas *p53* was mutated in 65% of these patients. Similarly, *c-Myc* and *FAK* were amplified or upregulated in 44 and 43%, respectively, of 594 patients in the TCGA Firehouse Legacy, while *p53* was mutated in 49% of these patients (Figure 3A). This finding suggests that c-Myc and FAK are potentially important drug targets for ovarian cancer therapy, a conclusion supported by the current clinical trials of several c-Myc or FAK inhibitors. In this study, we found that CT has a potency in inhibiting both c-Myc and FAK expression that was comparable to that of the c-Myc inhibitor 10058-F4 (Bashash et al., 2019). To examine how CT regulates c-Myc and FAK, we treated OVCAR3 and HEY A8 cells with different doses of CT or 10058-F4 for 24 h and monitored c-Myc and FAK expression by immunoblotting. CT mediated c-Myc and FAK inhibition in a dose-dependent manner with significant inhibition manifesting at the low (10–20 μM) doses (Figure 3B). CT or 10058-F4 applied at 10 μM inhibited expression of both c-Myc and FAK in a time-dependent manner, with a significant reduction detectable at 24 or 48 h in OVCAR3 and HEY A8 cells (Figure 3C).

### CT or knockdown of c-Myc attenuates RGD-induced FAK activation in ovarian cancer cells

**FIGURE 4:**
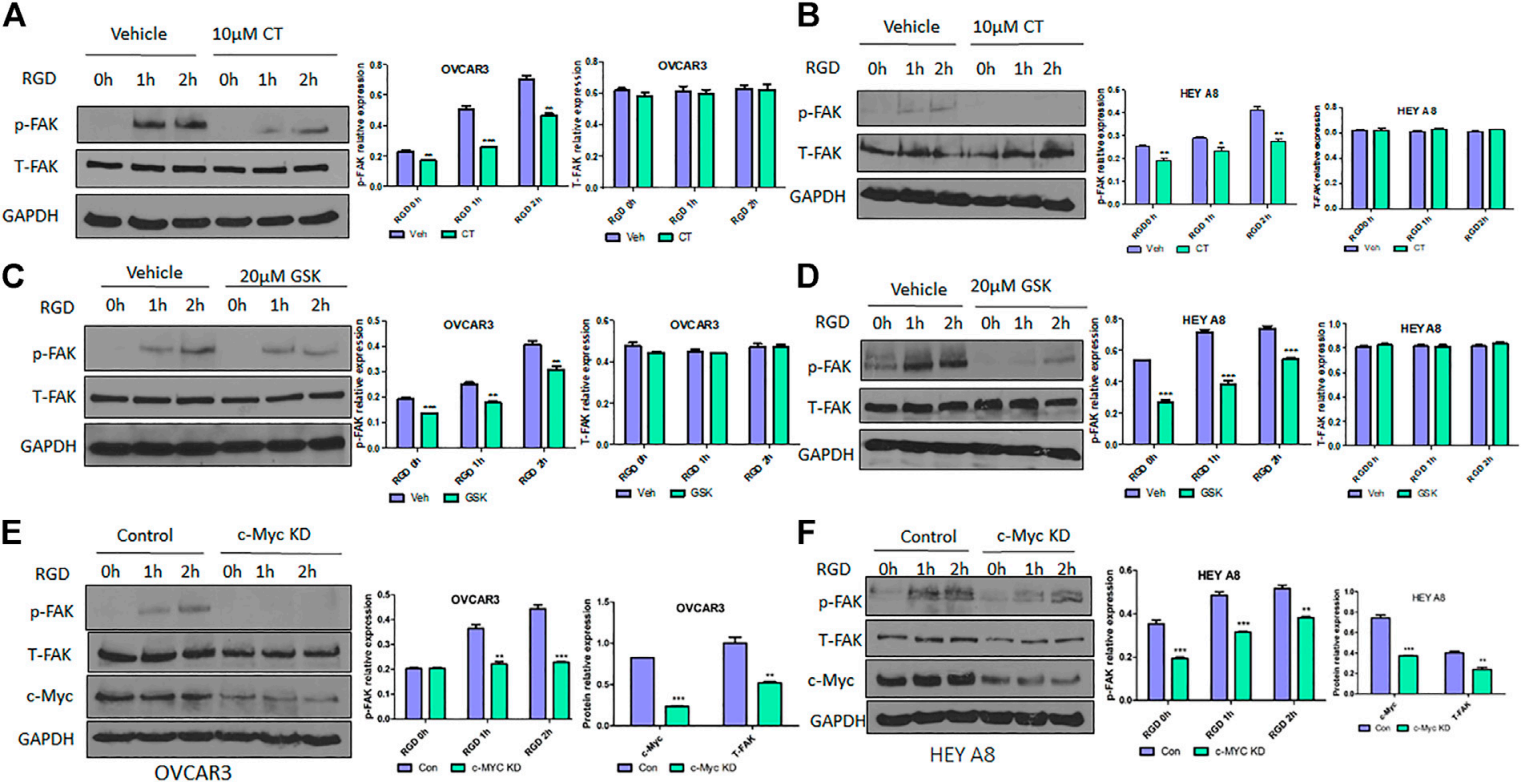
CT or genetic KD of c-Myc attenuates RGD-induced FAK activation in ovarian cancer cells. **(A,B)** Immunoblot and densitometry analysis of p-FAK and total FAK after treatment with 10 µM CT for 8 h and then 20 μM RGD for different time periods in OVCAR3 **(A)** and HEY A8 **(B)** cells. **(C,D)** Immunoblot and densitometry analysis of p-FAK and total FAK after treatment with 20 µM GSK2256098 for 8 h and then 20 µM RGD for different time periods in OVCAR3 **(C)** and HEY A8 **(D)** cells. **(E,F)** Immunoblot and densitometry analysis of p-FAK, total FAK, and c-Myc expression in c-Myc KD and control OVCAR3 **(E)** and HEY A8 **(F)** cells after treatment with 20 µM RGD for different time periods. Data are presented as mean values ±SD. *p < 0.05, **p < 0.01, ***p < 0.001.

Because CT mediated inhibition of c-Myc and FAK occurred after 12 h treatment, we assessed whether CT was able to block FAK activation at earlier time points. OVCAR3 and HEY A8 cells were treated with 10 μM CT or 20 μM FAK inhibitor GSK2256098 (Brown et al., 2018; Mak et al., 2019) for 8 h. Subsequently the FAK pathway was activated at different time points using 20 µM RGD (Feldkoren et al., 2017), a small peptide ligand of the integrin receptor. CT attenuated RGD-induced FAK activation but failed to induce complete FAK protein degradation at 8 h (Figures 4A,B). These effects were comparable to that of the FAK kinase inhibitor GSK2256098 in both cell lines (Figures 4C,D). We further tested whether a genetic approach with knockdown (KD) of c-Myc would phenocopy the pharmacological attenuation of FAK signaling. We established stable OVCAR3 and HEY A8 cell lines with c-Myc KD using a lentiviral CRISPR/Cas9 nickase vector and treated these cells with 20 µM RGD for varying times. RGD activated FAK phosphorylation in control OVCAR3 and HEY 8 cells, but activation was significantly attenuated in c-Myc KD cells with reduced c-Myc and FAK expression (Figures 4E,F). Overall, the attenuation of FAK signaling and FAK inhibition in c-Myc KD cells was consistent with the effects of CT in both cell lines, indicating that crosstalk between c-Myc and the FAK pathway could be suppressed by CT treatment.

### CT is similarly effective as the c-Myc inhibitor 10058-F4 in sensitizing ovarian cancer cell responses to chemotherapy-induced apoptosis

**FIGURE 5:**
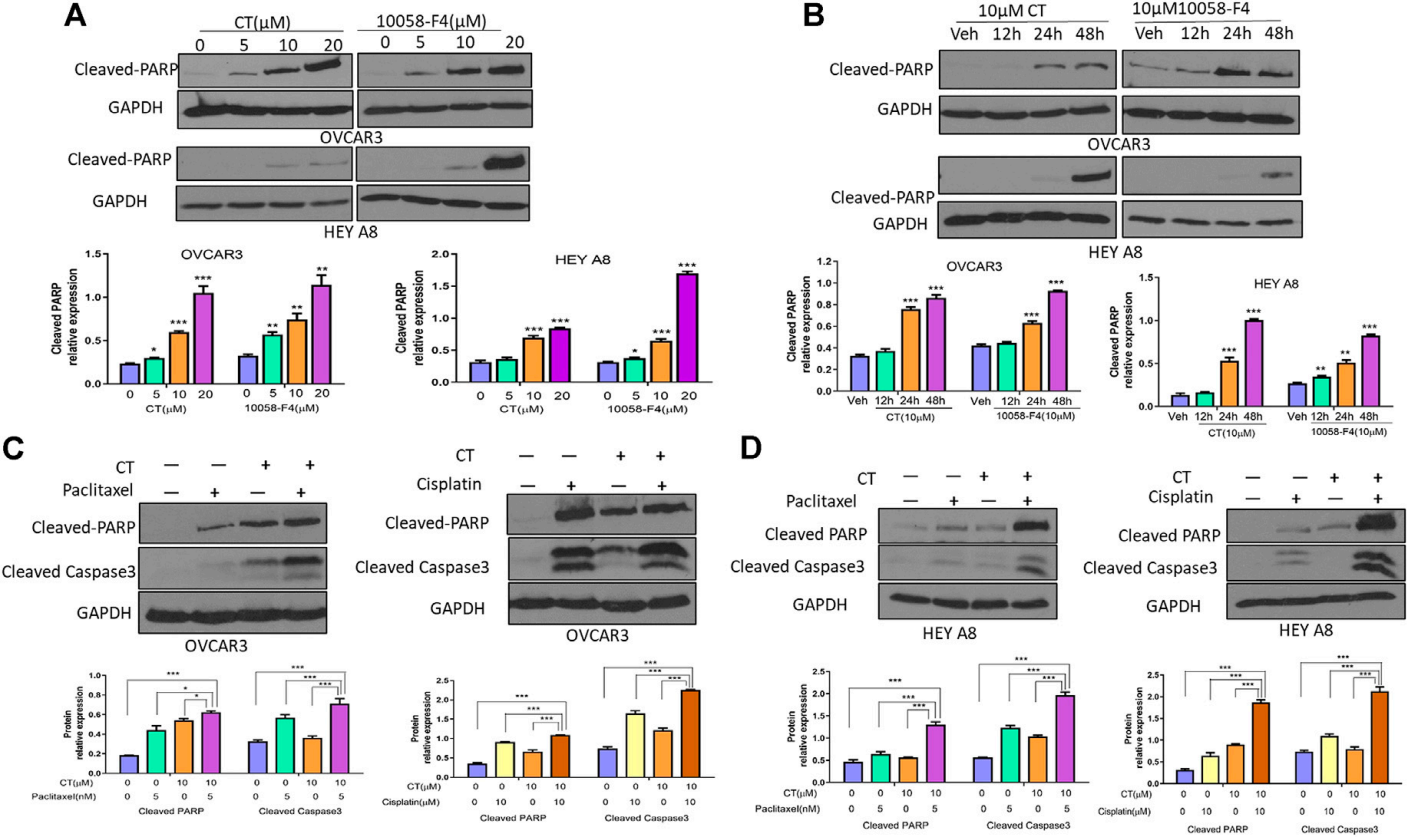
CT sensitizes chemotherapy drug induced apoptosis in ovarian cancer cells. **(A,B)** Immunoblot and densitometry analysis of apoptosis in OVCAR3 and HEY A8 cells by examining cleaved-PARP after treatment with **(A)** different doses of CT or 10058-F4 for 24 h or **(B)** the same dose of CT or 10058-F4 (10 µM) at different time points. **(C,D)** Immunoblot and densitometry analysis of apoptosis in OVCAR3 **(C)** and HEY A8 **(D)** cells by detection of cleaved-PARP and cleaved-caspase3 after 24 h treatment with CT alone or together with paclitaxel and cisplatin. One representative immunoblot from three similar independent experiments is shown. The expression of cleaved-PARP and cleaved-caspase3 were compared between cells treated with CT, 10058-F4, or vehicle. Data are presented as mean values ±SD. *p < 0.05, **p < 0.01, ***p < 0.001.

Because CT targets c-Myc and FAK in ovarian cancer cells, we hypothesized that this drug might induce apoptosis by functioning like a c-Myc inhibitor (Sheikh-Zeineddini et al., 2020). We examined the antitumor activity of CT in ovarian cancer cells and compared it with c-Myc inhibitor 10058-F4 by analyzing apoptosis as indicated by the levels of cleaved poly (ADP-ribose) polymerase (PARP) and caspase3 detected by immunoblotting. CT was not cytotoxic at 10 or 20 µM concentration in ovarian cancer cells (Supplementary Figures S1A,B). Therefore, we treated OVCAR3 and HEY A8 cells for 24 h with CT or 10058-F4 using concentrations that ranged from 5 to 20 µM. CT induced significant cleavage of PARP that was comparable to the cleavage elicited by 10058-F4 over the same concentration range in both cell lines. We detected only low levels of cleaved-PARP at the 5 µM dose in HEY A8 cells (Figure 5A). We also treated OVCAR3 and HEY A8 cells with 10 μM CT or 10058-F4 for different times. Both CT and 10058-F4 induced significant increase in PARP cleavage at 24 or 48 h time points in OVCAR3 cells but this response was delayed at 48 h in HEY A8 cells. This indicated that HEY A8 cells were more resistant to CT and 10058-F4-induced apoptosis than were OVCAR3 cells (Figure 5B).

To explore the potential therapeutic applicability of CT, we examined whether it displays a synergistic effect in inducing cell apoptosis when CT was administrated in combination with other chemotherapeutic drugs. OVCAR3 and HEY A8 cells were treated with CT and two commonly used chemotherapy drugs, paclitaxel and cisplatin. Compared to vehicle, 10 µM CT enhanced the efficacy of both paclitaxel (5 nM) and cisplatin (10 µM), as shown by the increased levels of cleaved PARP and caspase-3 (Figures 5C,D). We also examined apoptosis by performing immunofluorescent staining using the antibody that recognizes cleaved caspase-3. Apoptotic cell nuclei were detected in significantly higher numbers in cells treated with a combination of paclitaxel and CT or cisplatin and CT, relative to cells treated with CT, paclitaxel, or cisplatin alone (Supplementary Figures S2A,B). Our data indicated that CT enhanced the apoptosis of ovarian cancer cells induced by treatment with paclitaxel or cisplatin.

### CT degraded c-myc through increased ubiquitination by disrupting the interaction between max and c-myc in ovarian cancer cells

**FIGURE 6:**
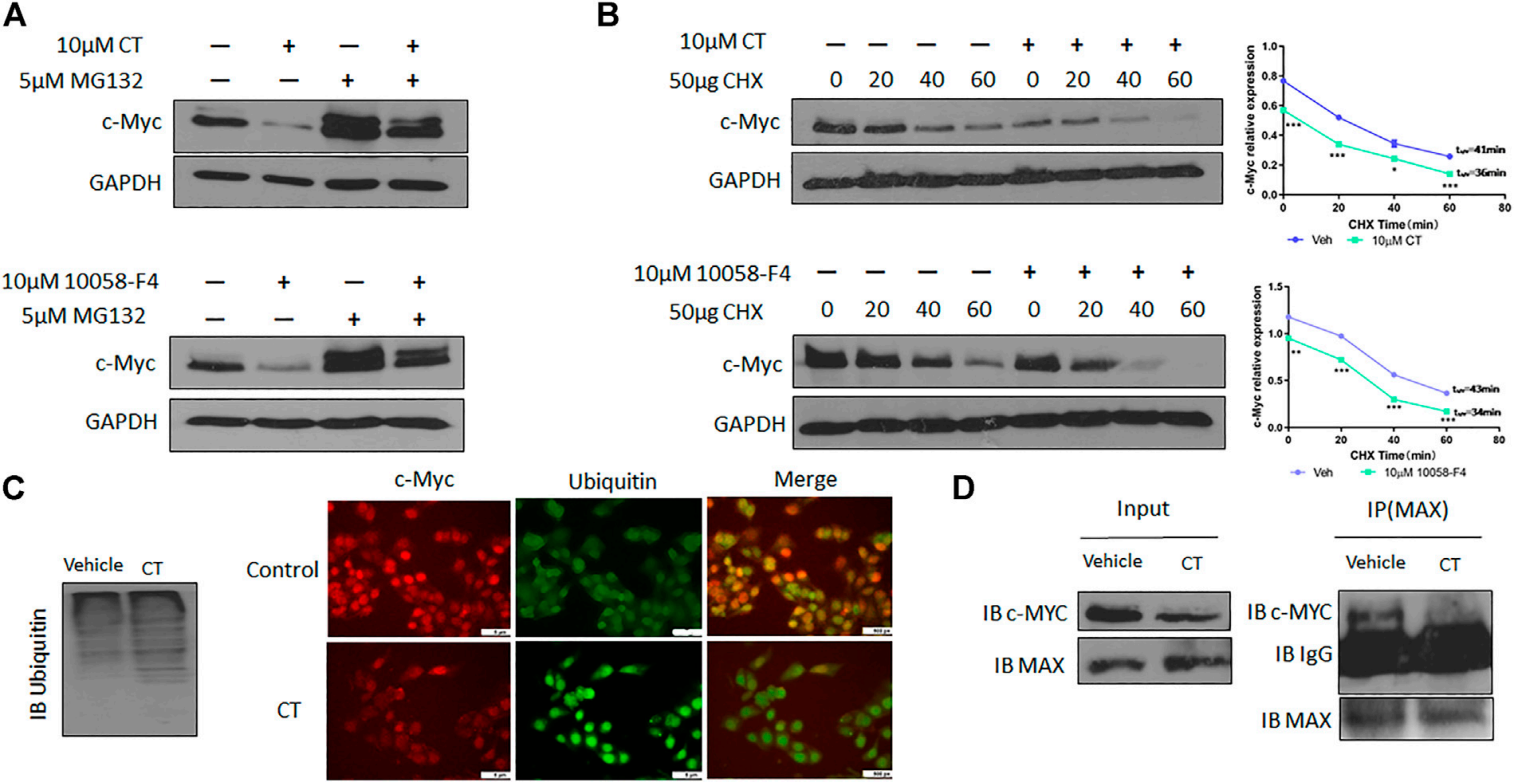
CT degrades c-Myc through ubiquitination pathway. **(A)** c-Myc expression in OVCAR3 cells treated with 10 μM CT or 10058-F4 with or without the proteasome inhibitor MG132 was determined by immunoblot. **(B)** OVCAR3 cells were pretreated with 10 µM CT or DMSO for 24 h, followed by 50 μg/ml CHX treatment and c-Myc expression was detected by immunoblot. **(C)** Immunoblot analysis of ubiquitination in OVCAR3 cells treated with vehicle or CT and immunofluorescent staining of c-Myc (Red) and ubiquitin (green) in OVCAR3 cells 24 h after treatment with CT or vehicle treatment. Scale bar, 5 μM. **(D)** Co-immunoprecipitation analysis using anti-MAX antibody in OVCAR3 cells treated with CT or vehicle for 24 h. Data are presented as mean values ±SD. (*p < 0.05, **p < 0.01, ***p < 0.001).

To understand the molecular mechanisms underlying CT-mediated c-Myc degradation, we treated OVCAR3 cells with the proteasome inhibitor MG132 (5 µM). MG132 treatment blocked the degradation of c-Myc induced by CT to an extent that was comparable to that induced by 10058-F4 (Figure 6A). We further assessed the stability of c-Myc using a cycloheximide chase assay and found that the half-life of c-Myc was significantly reduced from 41 to 36 min by CT treatment in OVCAR3 cells, whereas the control drug 10058-F4 reduced it from 43 min to 34 min. These results suggest that CT promoted c-Myc proteasomal degradation in a manner similar to that of the c-Myc inhibitor 10058-F4 (Figure 6B).

**FIGURE 7:**
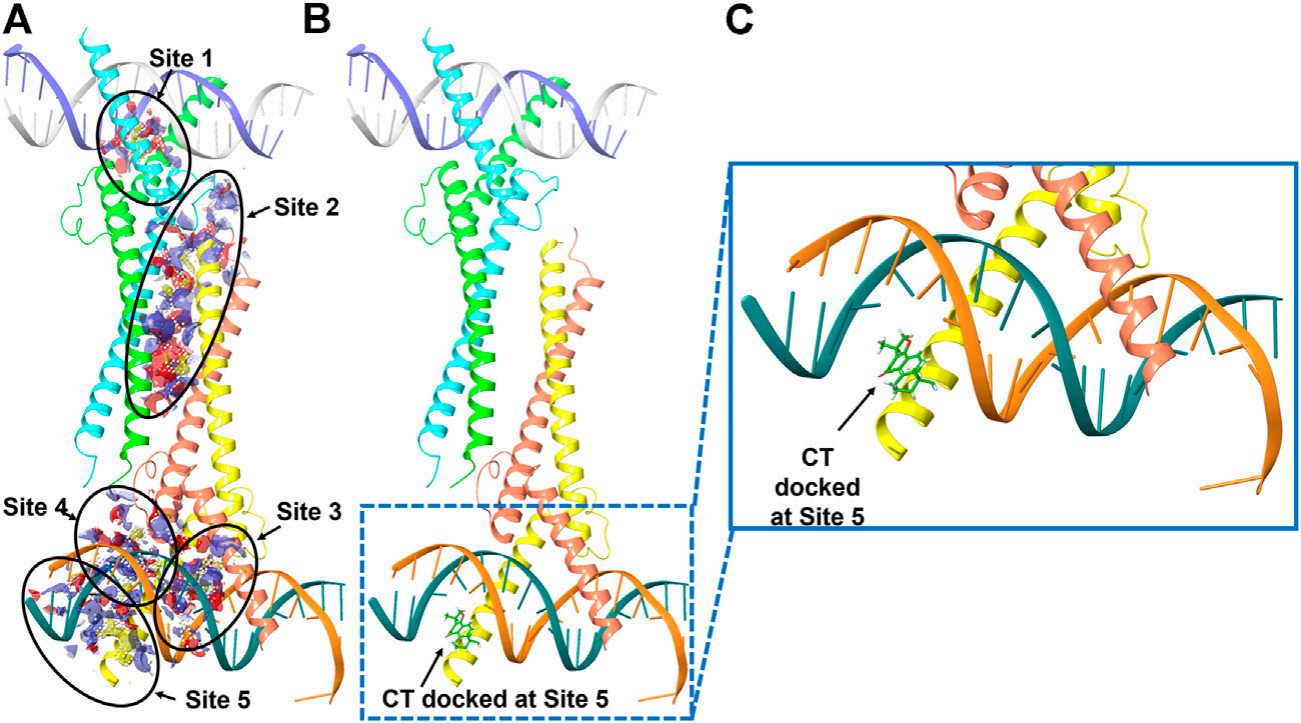
Molecular modeling of CT and c-Myc/Max. **(A)** Five predicted potential binding sites in the Myc-Max-DNA complex (PDB ID 1NKP). **(B)** Binding pose of CT docked in the complex that has the best docking score. **(C)** Zoom-in view of the binding pose. Subunits of Myc proto-oncogene protein are labeled in green and orange. Subunits of Max protein are labeled in cyan and yellow.

We therefore examined whether c-Myc degradation occurs through the ubiquitination pathway in ovarian cancer cells. We treated OVCAR3 cells with 10 μM CT for 24 h and detected ubiquitination by immunoblot using an anti-ubiquitin antibody. Levels of c-Myc ubiquitination were increased in CT-treated cells relative to vehicle-treated control cells (Figure 6C). Induction of ubiquitination by CT was also validated by fluorescent staining, which was stronger in CT-treated than vehicle treated cells (Figure 6C). We further examined whether CT-mediated ubiquitination of c-Myc by disrupting the interaction between c-Myc and Max, which is required for c-Myc stability. Thus, we treated OVCAR3 cells with 10 μM CT for 24 h, co-immunoprecipitated with antibodies specific for Max, and the immunocomplex was blotted with c-Myc antibodies. As we expected, we were unable to detect c-Myc expression in CT-treated cells using this method but detected c-Myc expression in vehicle treated cells, suggesting that CT disrupted the interaction between c-Myc and Max (Figure 6D). To detect CT induced c-Myc polyubiquitination, we treated OVCAR3 for 6 h with 10 µM CT and 5 µM MG132, the protein complex was immunoprecipitated with c-Myc antibodies and detected by immunoblot with antibodies specific for ubiquitin and c-Myc antibody. The ubiquitinated c-Myc was clearly detected by ubiquitin antibodies in OVCAR3 cells treated with MG132 or MG132 and CT (Supplementary Figure S3). Then we used dock modeling to predict how CT binds to c-Myc/Max. The crystal structure of Myc-Max recognizing DNA was solved by Nair et al., in 2003 (PDB ID: 1NKP) (Nair and Burley, 2003; Mustata et al., 2009; Jung et al., 2015). There were reports on small molecules inhibitors of c-Myc that exert their functions through direct interaction with the complex. We used Schrödinger Maestro and performed computational modeling. Through SiteMap wizard, five possible binding sites were predicted (Figure 7A). We docked CT into those five pockets, respectively, and yielded the best docking score of -5.6 at site 5. The predicted binding mode is shown in Figures 7B,C, indicating a possible mechanism of action of CT. These data suggest that CT degraded c-Myc by disrupting its stability via the proteasomal degradation pathway.

We also determined expression of epithelial to mesenchymal transition (EMT) markers in OVCAR3 cells after CT treatment for 24 h. We found that CT inhibited the expression of the mesenchymal markers N-cadherin and β-catenin whereas it increased the expression of epithelial markers E-cadherin (Supplementary Figure S4), indicating that CT inhibits EMT in ovarian cancer cells.

### CT inhibits primary ovarian tumor growth and metastasis *in vivo*

**FIGURE 8:**
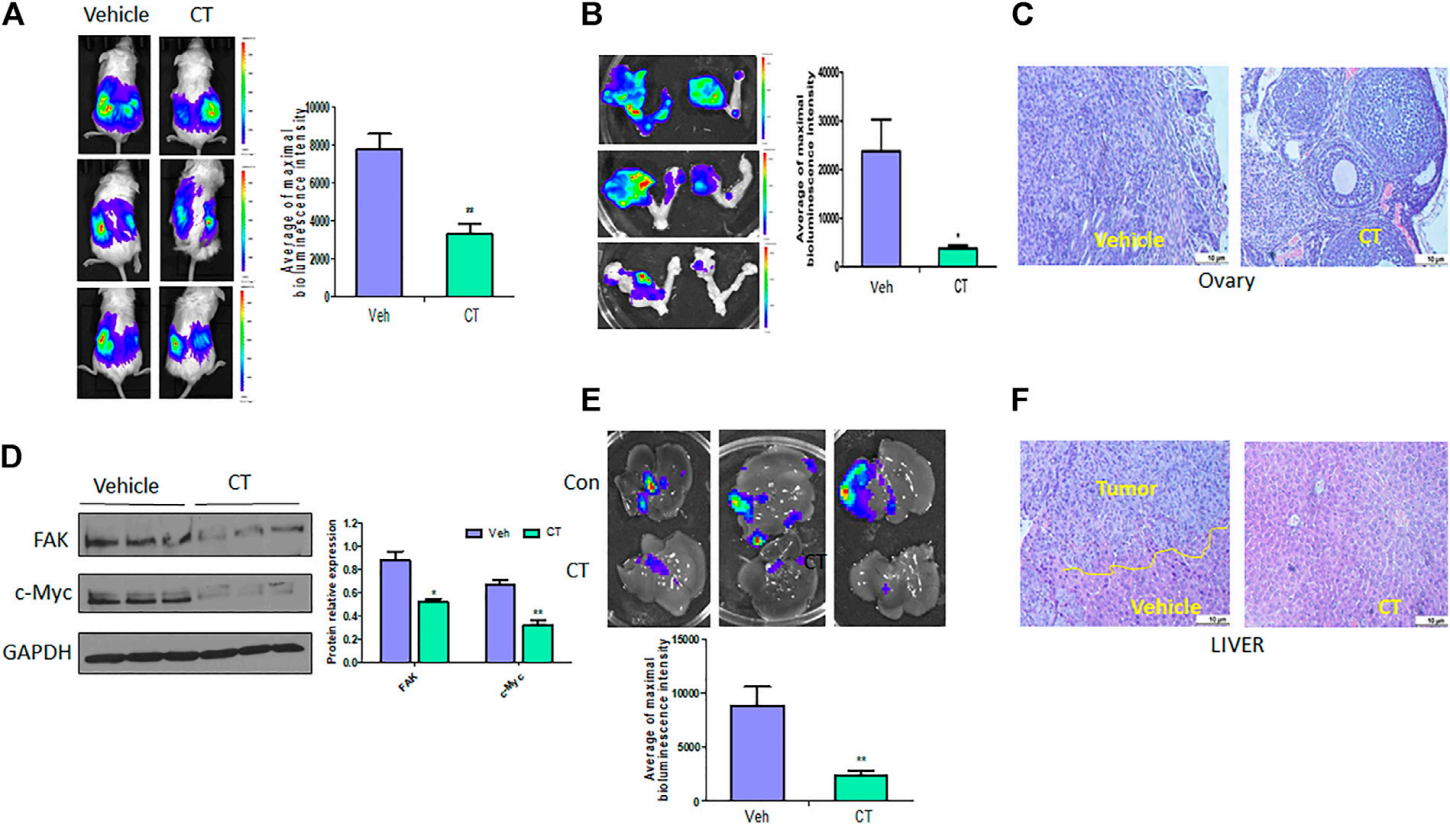
CT inhibits ovarian tumor growth and metastasis. **(A,B)** Live bioluminescence imaging of mice **(A)** and primary ovarian tumors **(B)** 3 weeks after CT or vehicle treatment of NSG mice intrabursally injected with WT HEY A8 cells. *n* = 5, *p < 0.05, **p < 0.01. **(C)** Sections of primary ovarian tumors were stained with HE. **(D)** Immunoblot and densitometry analysis of c-Myc and FAK expression in primary tumor from three different xenografted mice treated with CT or vehicle. *p < 0.05, **p < 0.01. **(E)** Metastatic tumors in liver of mice treated with CT or vehicle. **(F)** Sections of metastatic tumors in liver were stained with HE. *p < 0.05, **p < 0.01.

To test the efficacy of CT in ovarian tumor therapy, we injected WT HEY A8 cells intrabursally into NSG female mice and treated them i. p. With vehicle or CT (10 mg/kg bodyweight) for 3 weeks. Primary tumors in ovaries were significantly reduced in CT-treated mice compared to vehicle-treated mice, as shown by bioluminescence in live mice (Figure 8A), and in ovaries dissected from these mice (Figure 8B). Ovarian tumors were observed in vehicle-treated mice but were reduced in size in HE-stained ovaries from CT-treated mice (Figure 8C). We also examined the level of c-Myc and FAK in primary ovarian tumors using immunoblot analysis with antibodies specific for c-Myc or FAK. c-Myc and FAK were both significantly reduced in ovarian tumors in mice treated with CT, relative to those in control mice (Figure 8D). When we examined tumor metastasis in this animal model, we found that treatment with CT significantly inhibited tumor metastasis in multiple peritoneal organs including the liver, relative to vehicle-treated mice (Figures 8E,F). These results demonstrated that CT suppressed primary ovarian tumor growth and metastasis by inhibiting c-Myc and FAK.

## Discussion

CT as a natural compound with demonstrated antitumor activity in a variety of cancers. In this study, we demonstrated for the first time that CT inhibits HEY A8 ovarian tumor growth and metastasis in an orthotopic mouse model. In particular, we defined a novel molecular mechanism underlying CT’s antitumor activity by demonstrating degradation of c-Myc through ubiquitination. We found that CT also attenuated FAK signaling at an early time point and inhibited FAK activities at a later time point. Although we choose two different cell lines with different p53 status, we found no impact of p53 expression on CT treatment (data not shown).

CT induced apoptosis in HEY A8 and OVCAR3 cell lines. However, we found that the HEY A8 cell line was more resistant to CT- and 10058-F4-induced apoptosis than were OVCAR3 cells. HEY A8 cells were also more aggressively metastatic than OVCAR3 cells in our orthotopic mouse models. For this reason, we selected the HEY A8 cell line to test the therapeutic efficacy of CT. Although p53 is WT in HEY A8 cells, while it is mutated in OVCAR3 cells, our data supported the idea that the efficacy of CT was independent of the p53 status of these cell lines. CT displayed a synergistic effect in promoting the apoptosis induced by paclitaxel and cisplatin in these cells, suggesting that CT is a *bona fide* antitumor agent when applied in combination with these chemotherapeutic drugs. CT inhibited ovarian cancer cell proliferation at concentrations above 10 µM. Our experiments demonstrated that CT controls tumor cell growth by inhibiting proliferation, migration, and invasion, and induces apoptosis. In our orthotopic ovarian cancer mouse models, CT inhibited primary ovarian tumor growth and metastasis. From a therapeutic perspective, we note that CT enhances the efficacy of chemotherapeutic drugs used in the treatment of ovarian cancer (Figure 4). However, the potency of CT in overcoming chemoresistance using ovarian cancer cell lines that are resistant to chemotherapy drug resistant *in vitro* or in mouse models *in vivo* remains to be tested.

c-Myc and FAK were highly expressed in both of the cell lines we tested. Based on the aberrant expression of c-Myc and FAK in ovarian cancer, it is important to target both genes. Interestingly, we observed that CT degraded both c-Myc and FAK in ovarian cancer cells after 12 h treatment, which is comparable to the effects of the Myc inhibitor drug 10058-F4 (Figure 3). We detected the mRNA expression of c-Myc and FAK using quantitative RT-PCR (data not shown) but did not find any significant differences between cells treated with CT and those treated with vehicle, suggesting that c-Myc and FAK degradation may be regulated at the posttranscriptional level. MG132 inhibited CT-induced c-Myc degradation, indicating that it may induce degradation of c-Myc via the proteasomal degradation pathway. Indeed, our results showed that CT promoted ubiquitination in ovarian cancer cells. However, while CT did not affect the level of expression of the c-Myc binding partner Max, co-immunoprecipitation studies with Max-specific antibodies showed that CT disrupted the heterodimerization between Max and c-Myc. Our computational dock modeling also supports a direct interaction between CT and c-Myc/Max complex. All of the above results suggest that CT targets c-Myc by disrupting its stability.

Although we observed that CT degraded c-Myc and FAK in both cell lines regardless of their p53 status, it remains unclear whether CT treatment directly leads to FAK degradation or degrades only c-Myc, subsequently leading to a decrease in FAK. To answer this question, we knocked down (KD) c-Myc using lentiviral CRISPR/Cas9 nickase and found that KD of c-Myc resulted in reduction in FAK protein. This suggests that FAK expression may be regulated by c-Myc, and the reduction in our detection of FAK might be a result of c-Myc degradation. Previous studies showed that N-Myc transcriptionally regulated FAK expression by binding to the FAK promoter in neuroblastoma cells (Beierle et al., 2007). Therefore, it is plausible that c-Myc transcriptionally activates FAK expression, leading to FAK upregulation in ovarian cancer cells. We failed to detect a direct interaction between c-Myc and FAK using a co-IP assay, although FAK also functions as a scaffold protein that is separate from its kinase activity.

**FIGURE 9:**
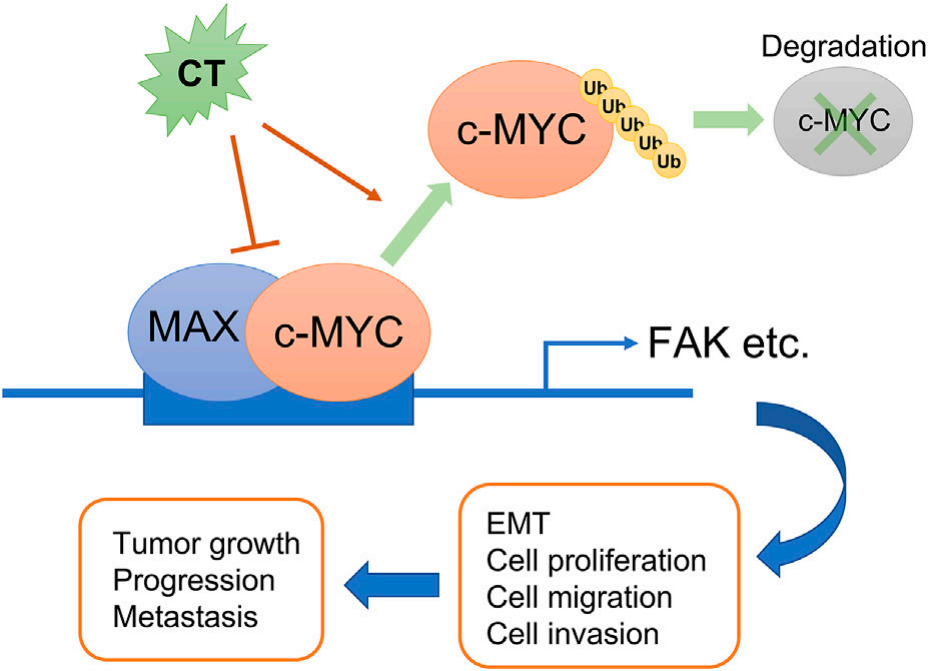
Schematic diagram summarizing the role of CT on ovarian cancer progression. CT inhibits ovarian tumor growth and metastasis via degradation of c-Myc by disrupting its stability and attenuating the FAK signaling pathway.

In conclusion, we demonstrated that CT is a potential antitumor drug candidate in ovarian cancer therapy not only because it improves the efficacy of other chemotherapeutic drugs but more importantly because it causes the degradation of c-Myc by disrupting its stability and attenuating the FAK signaling pathway (Figure 9).

## Data Availability

The original contributions presented in the study are included in the article/Supplementary Material, further inquiries can be directed to the corresponding authors.
