# Supplementary material for: Cryptotanshinone inhibits ovarian tumor growth and metastasis by degrading c-Myc and attenuating the FAK signaling pathway
Source: Front Cell Dev Biol. 2022 Sep 28;10:959518. doi: 10.3389/fcell.2022.959518 (PMC9554091; doi:10.3389/fcell.2022.959518)

Original data (Fig1-6 and 8, Fig S1, S2)

Figure 1

C

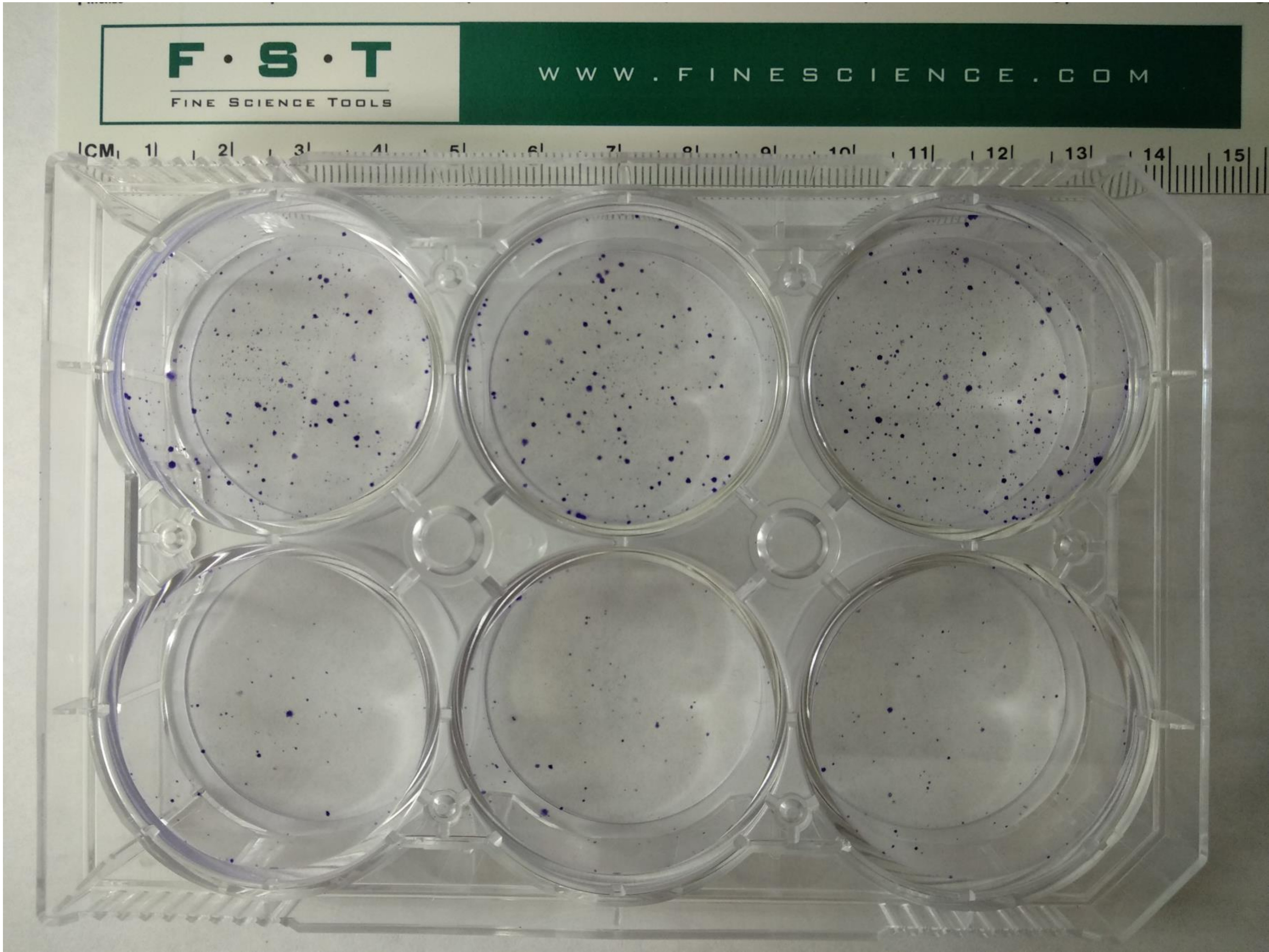
F·S·T
FINE SCIENCE TOOLS
WWW.FINESCIENCE.COM
CM 1 2 3 4 5 6 7 8 9 10 11 12 13 14 15

Figure 1

D

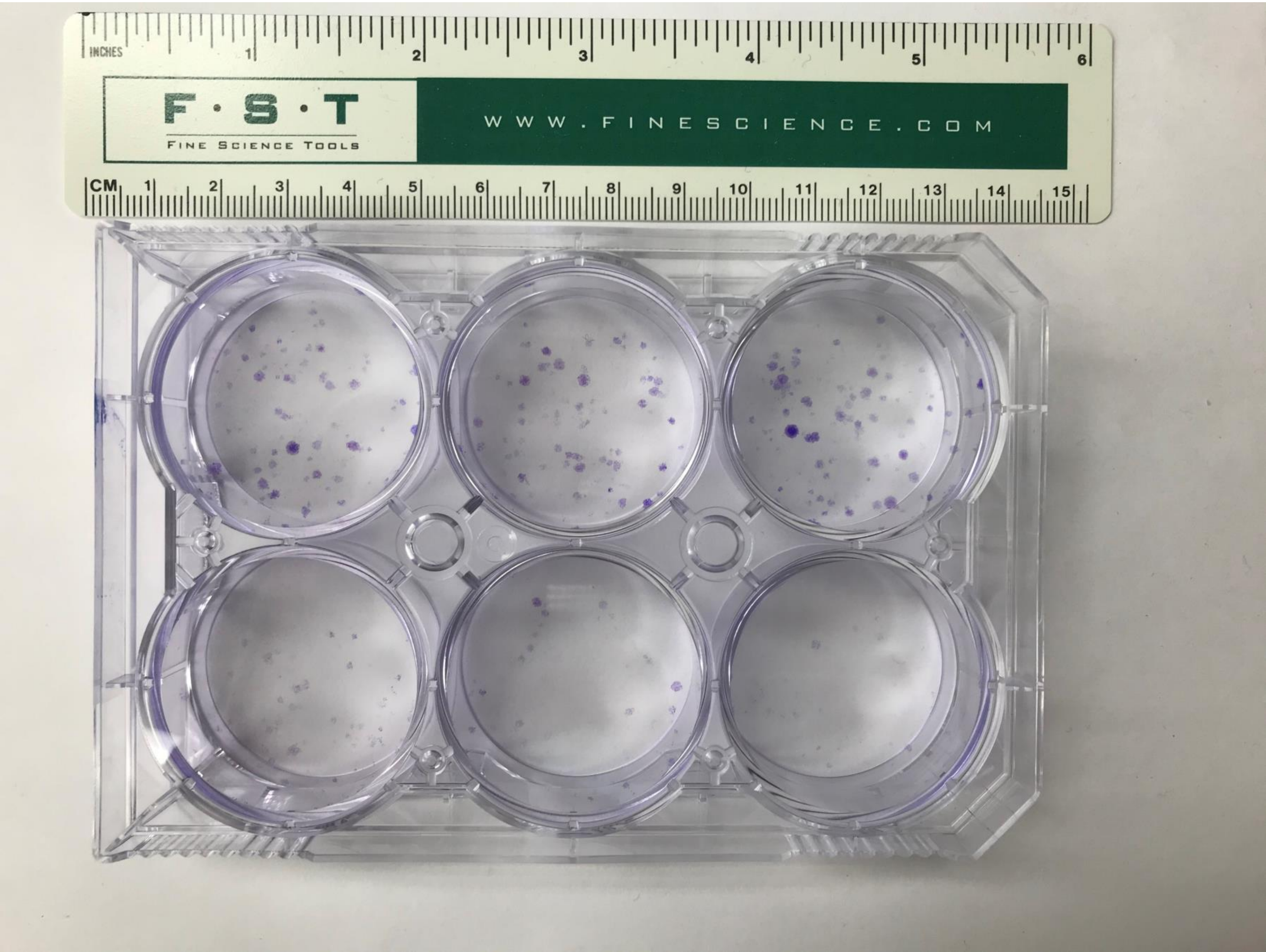
INCHES
1
2
3
4
5
6
F·S·T
FINE SCIENCE TOOLS
WWW.FINESCIENCE.COM
CM
1
2
3
4
5
6
7
8
9
10
11
12
13
14
15

Figure 2

A

OVCAR3 Migration

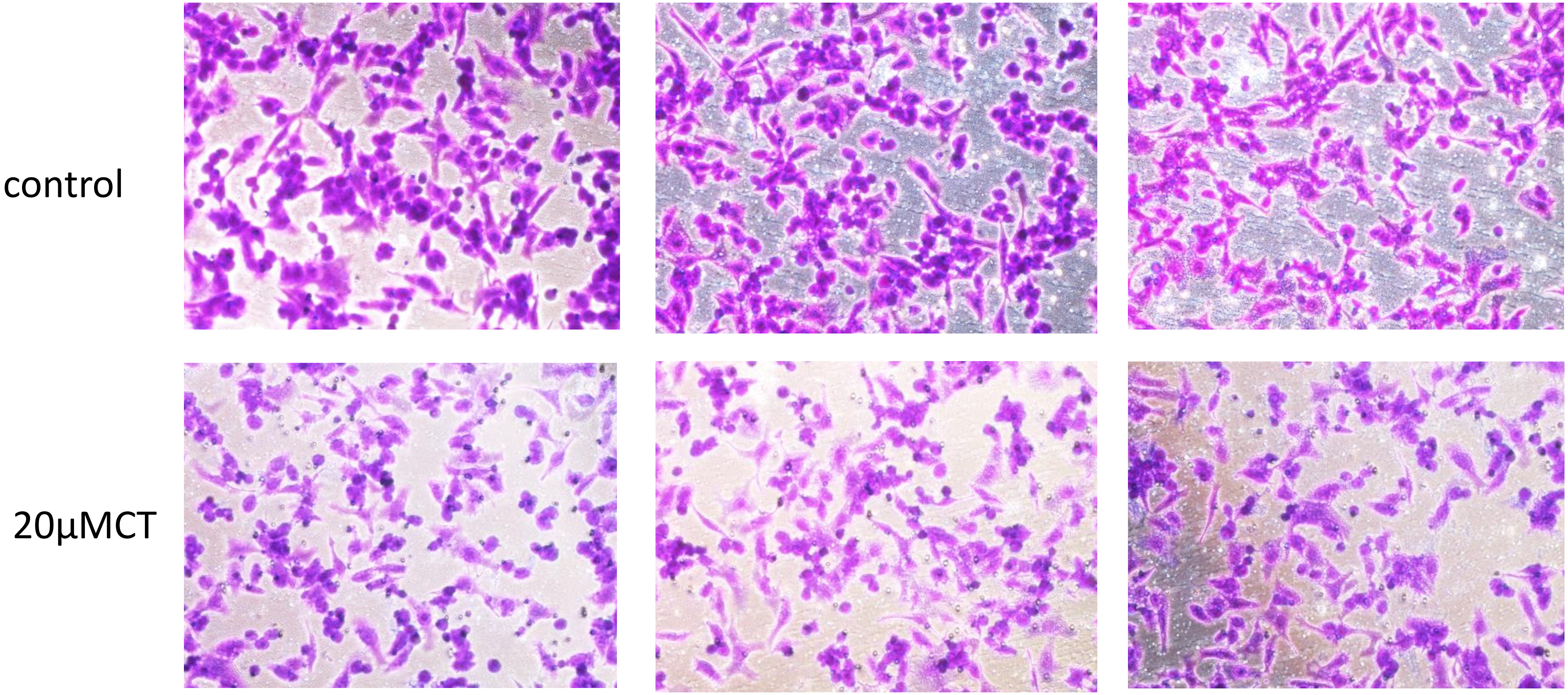

Figure 2

A HEY A8 Migration

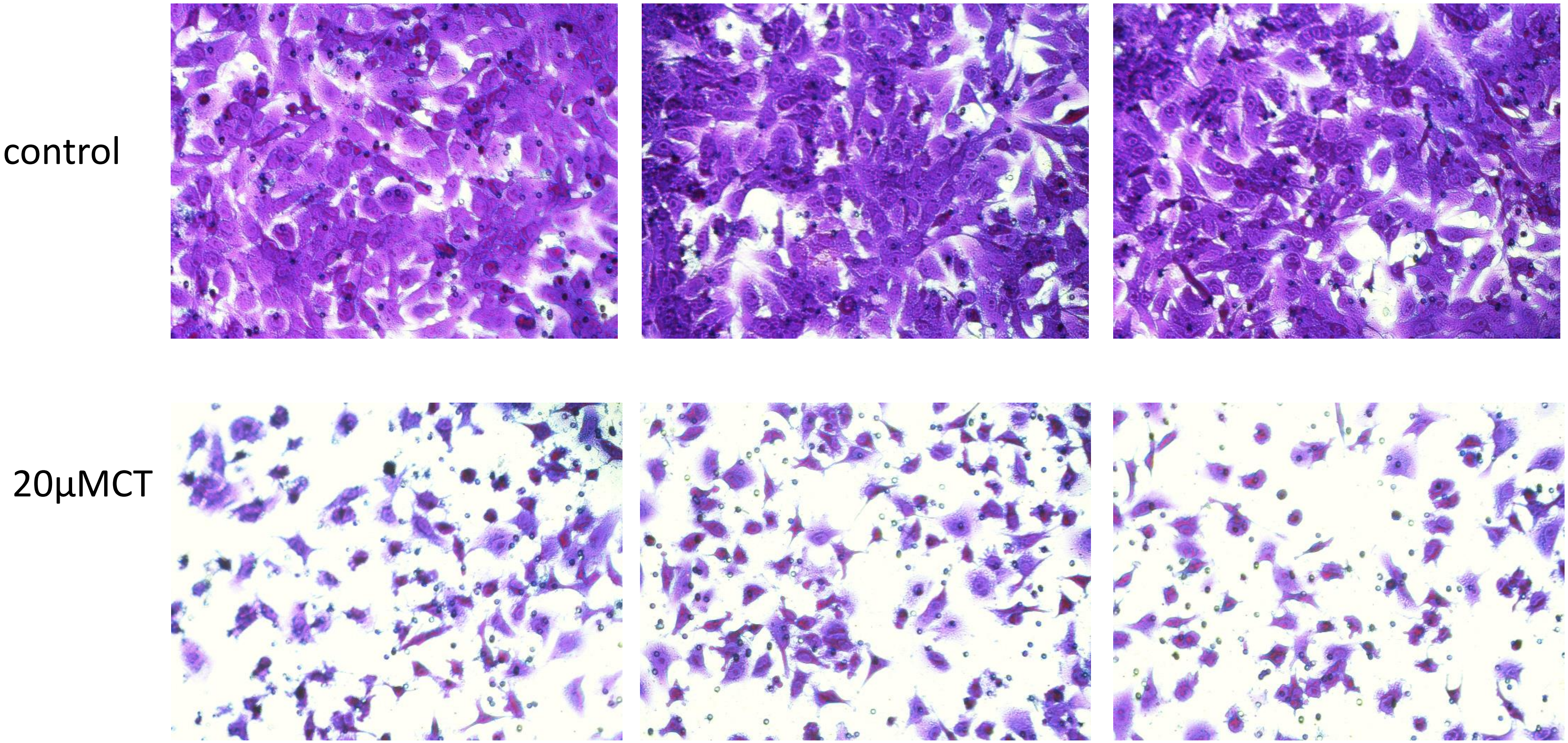

Figure 2

B OVCAR3 Invasion

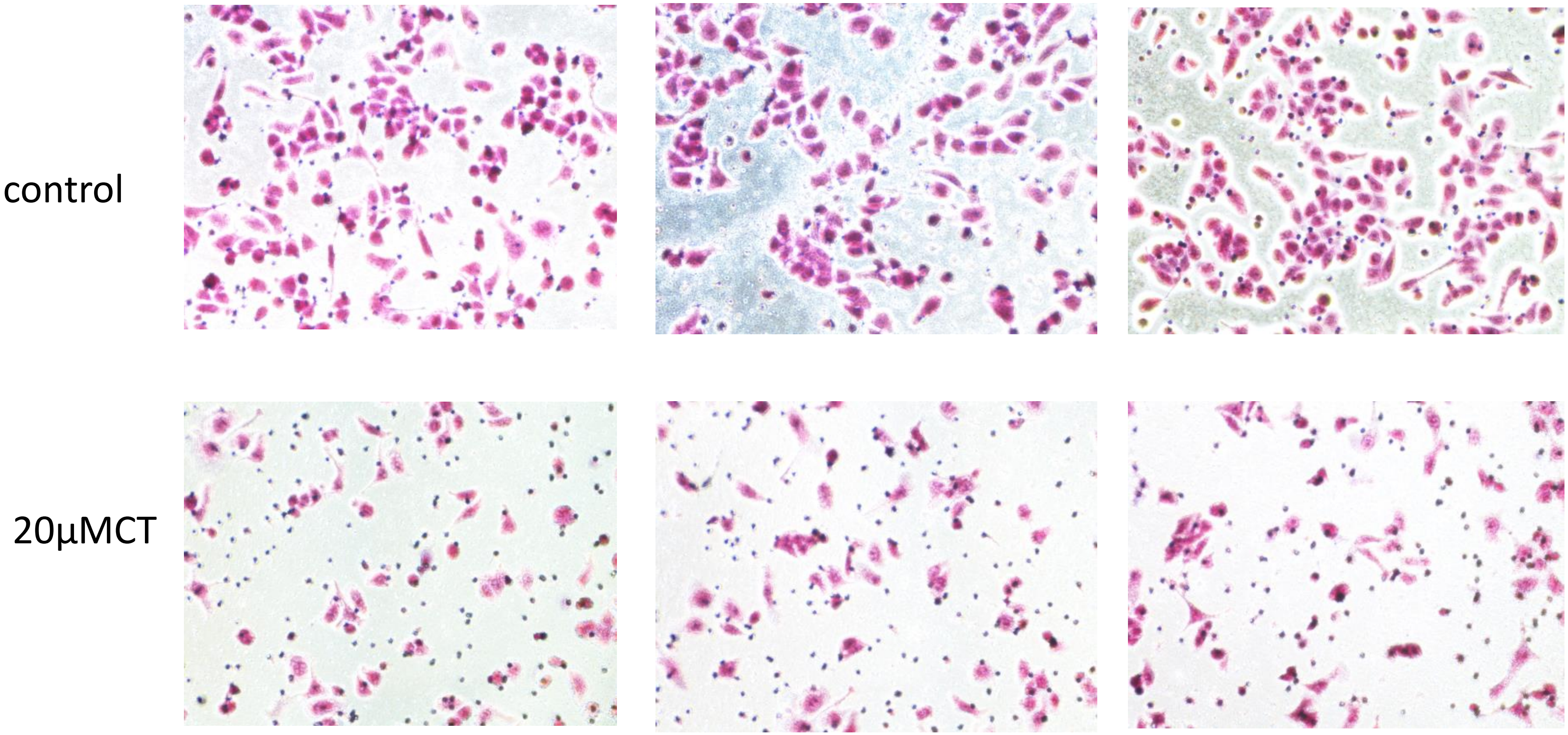

Figure 2

B

HEY A8 Invasion

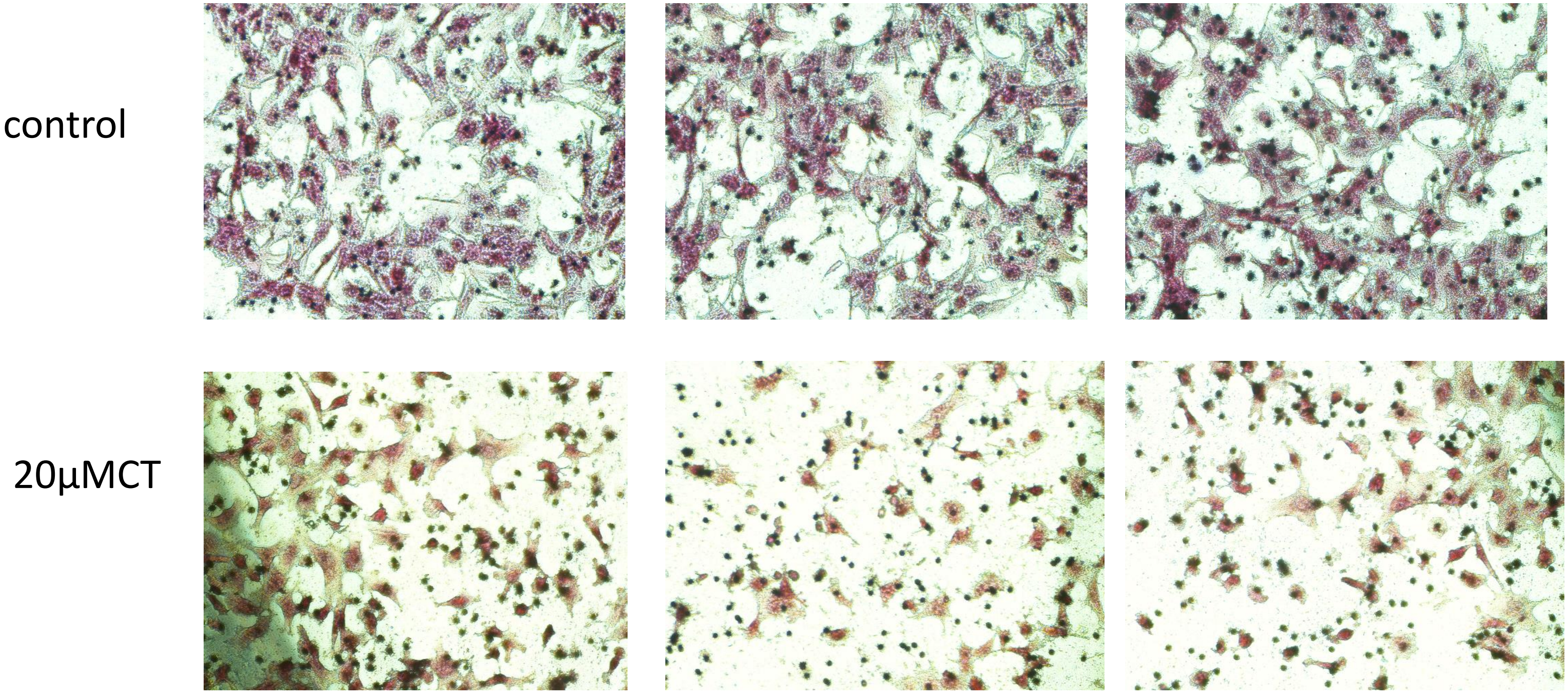

Figure 3

B

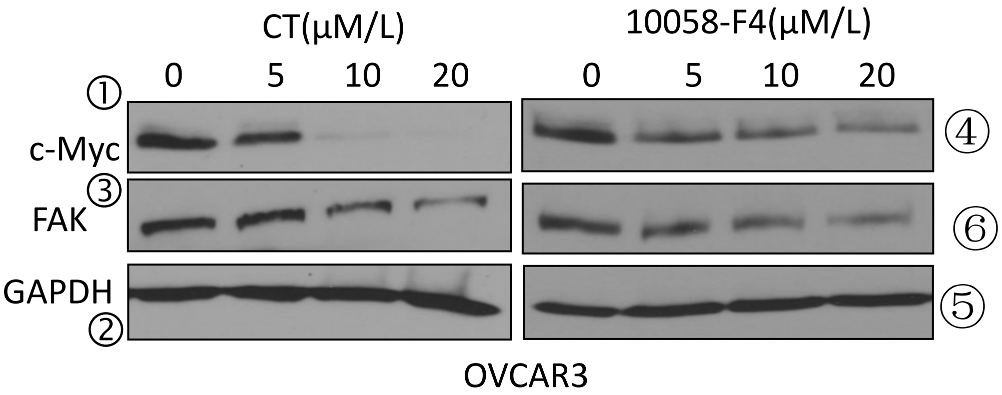
CT(μM/L)
0 5 10 20
10058-F4(μM/L)
0 5 10 20
①
c-Myc
③
FAK
GAPDH
②
④
⑥
⑤
OVCAR3

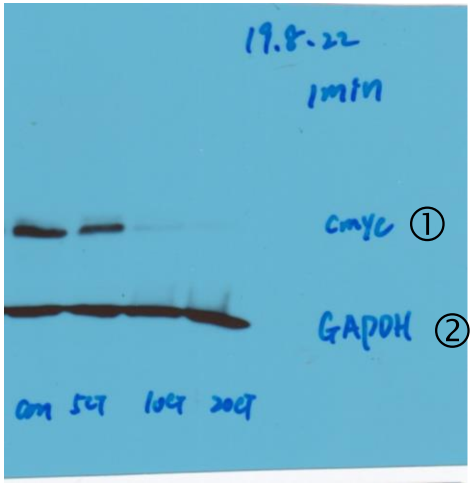
19.8.22
1min
Cmyc ①
GAPDH ②

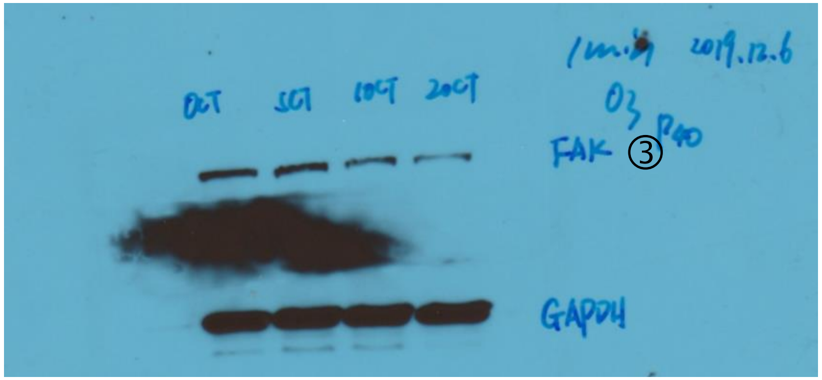
2019.12.6
FAK ③
GAPDH

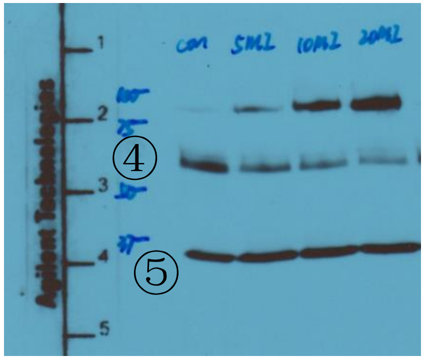
④
⑤

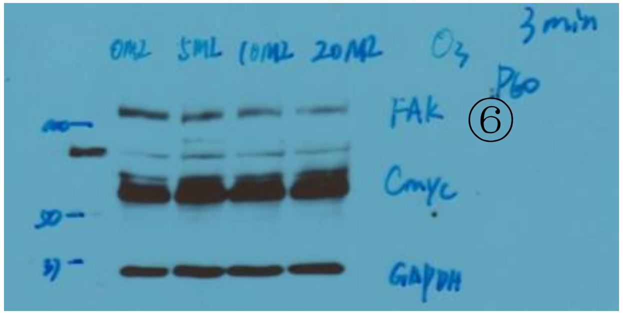
3min
FAK ⑥
Cmyc
GAPDH

Figure 3

B

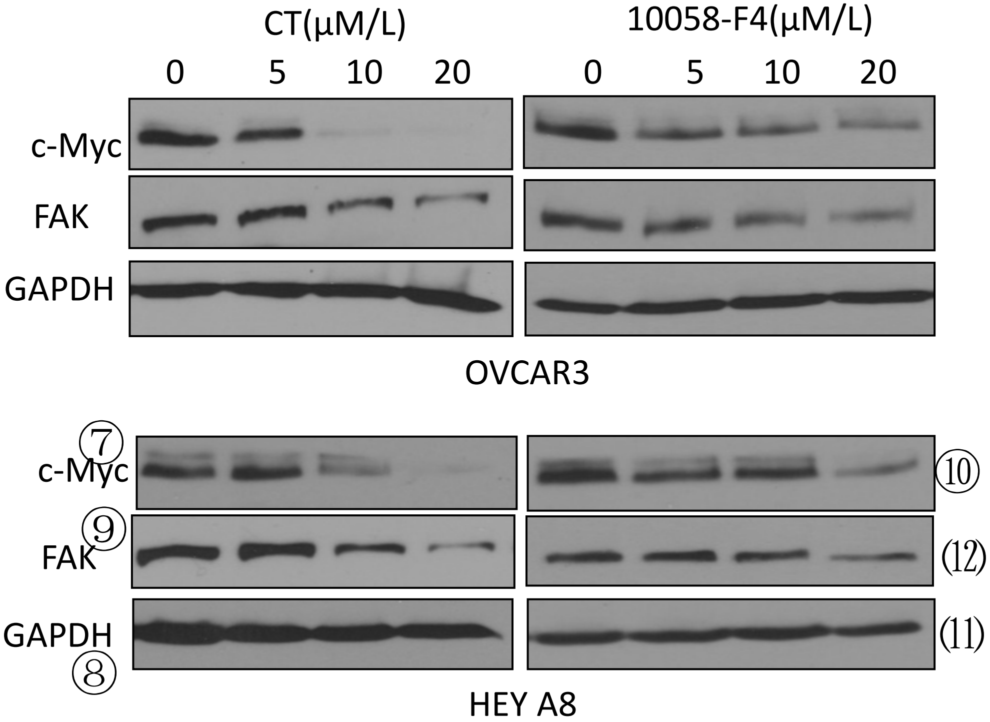
CT(μM/L)
0 5 10 20
10058-F4(μM/L)
0 5 10 20
c-Myc
FAK
GAPDH
OVCAR3
⑦ c-Myc ⑩
⑨ FAK (12)
GAPDH ⑧ (11)
HEY A8

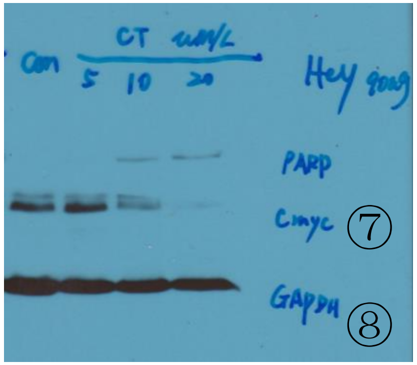
Con
CT
5 10 20
PARP
Cmyc ⑦
GAPDH ⑧

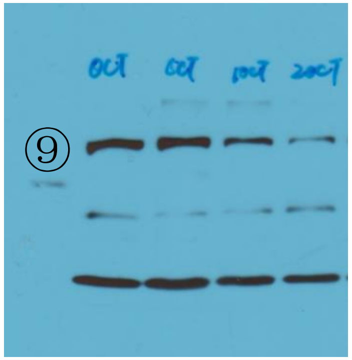
⑨

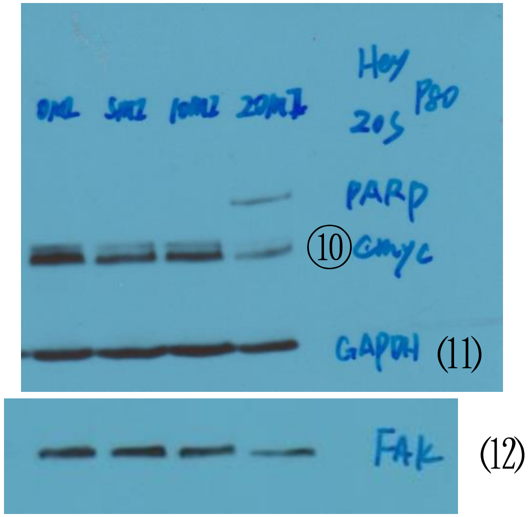
Hey
PARP
⑩ Cmyc
GAPDH (11)
FAK (12)

Figure 3

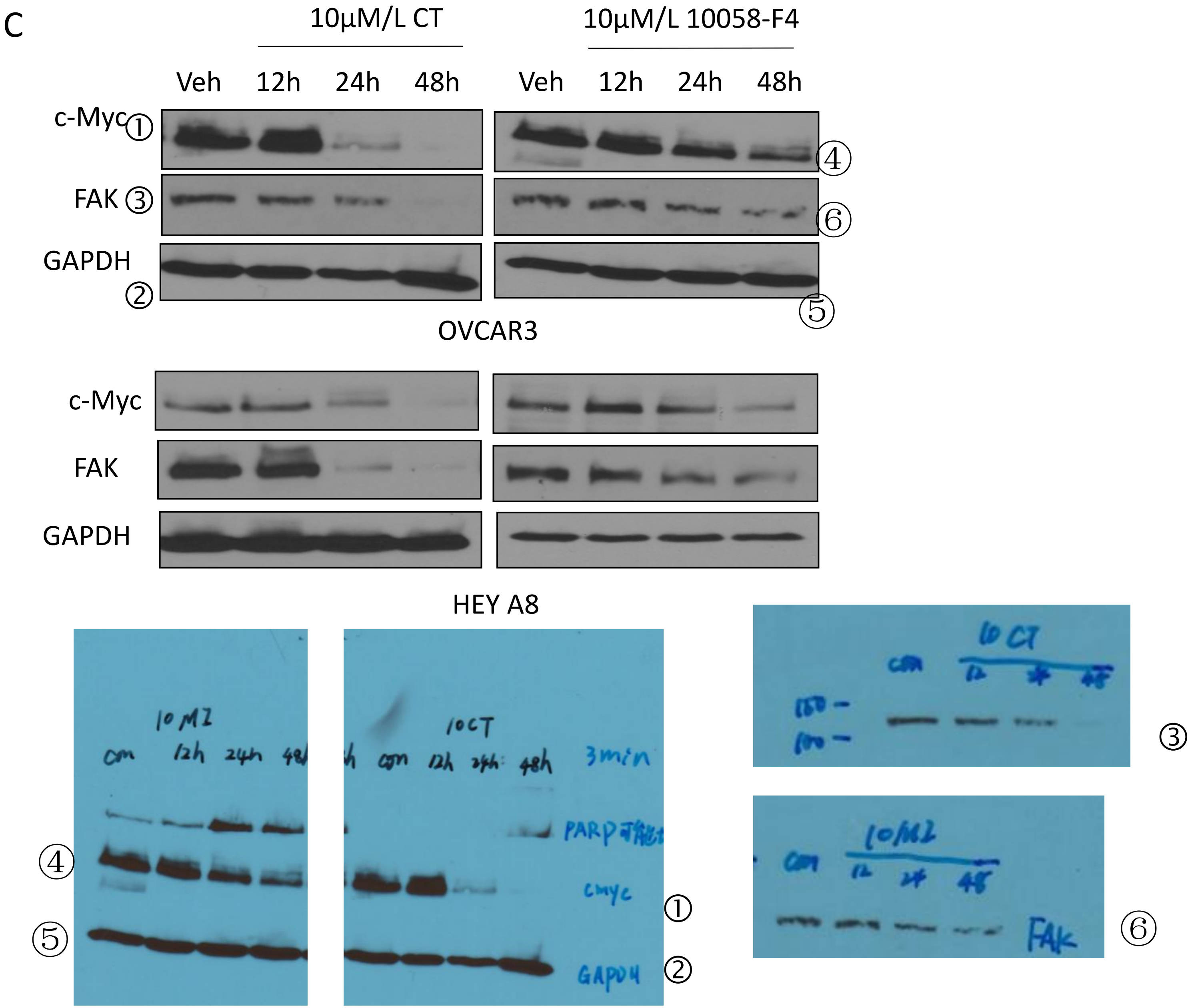

C
10μM/L CT
10μM/L 10058-F4
Veh
12h
24h
48h
Veh
12h
24h
48h
c-Myc
FAK
GAPDH
OVCAR3
c-Myc
FAK
GAPDH
HEY A8

Figure 3

C

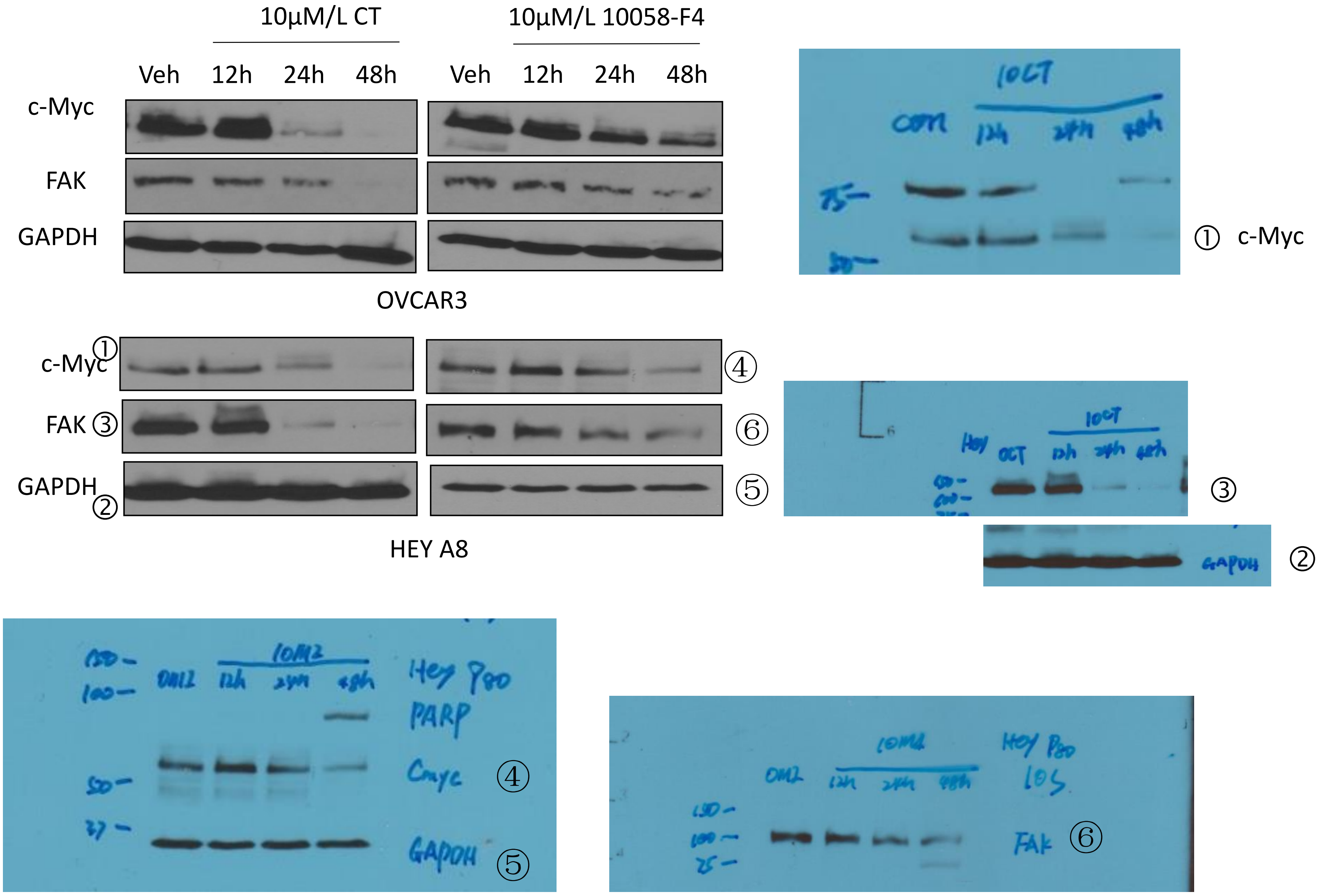
10μM/L CT
10μM/L 10058-F4
Veh
12h
24h
48h
Veh
12h
24h
48h
c-Myc
FAK
GAPDH
OVCAR3
c-Myc ①
FAK ③
GAPDH ②
④
⑥
⑤
HEY A8
① c-Myc
③
②
④
⑤
⑥

Figure 4

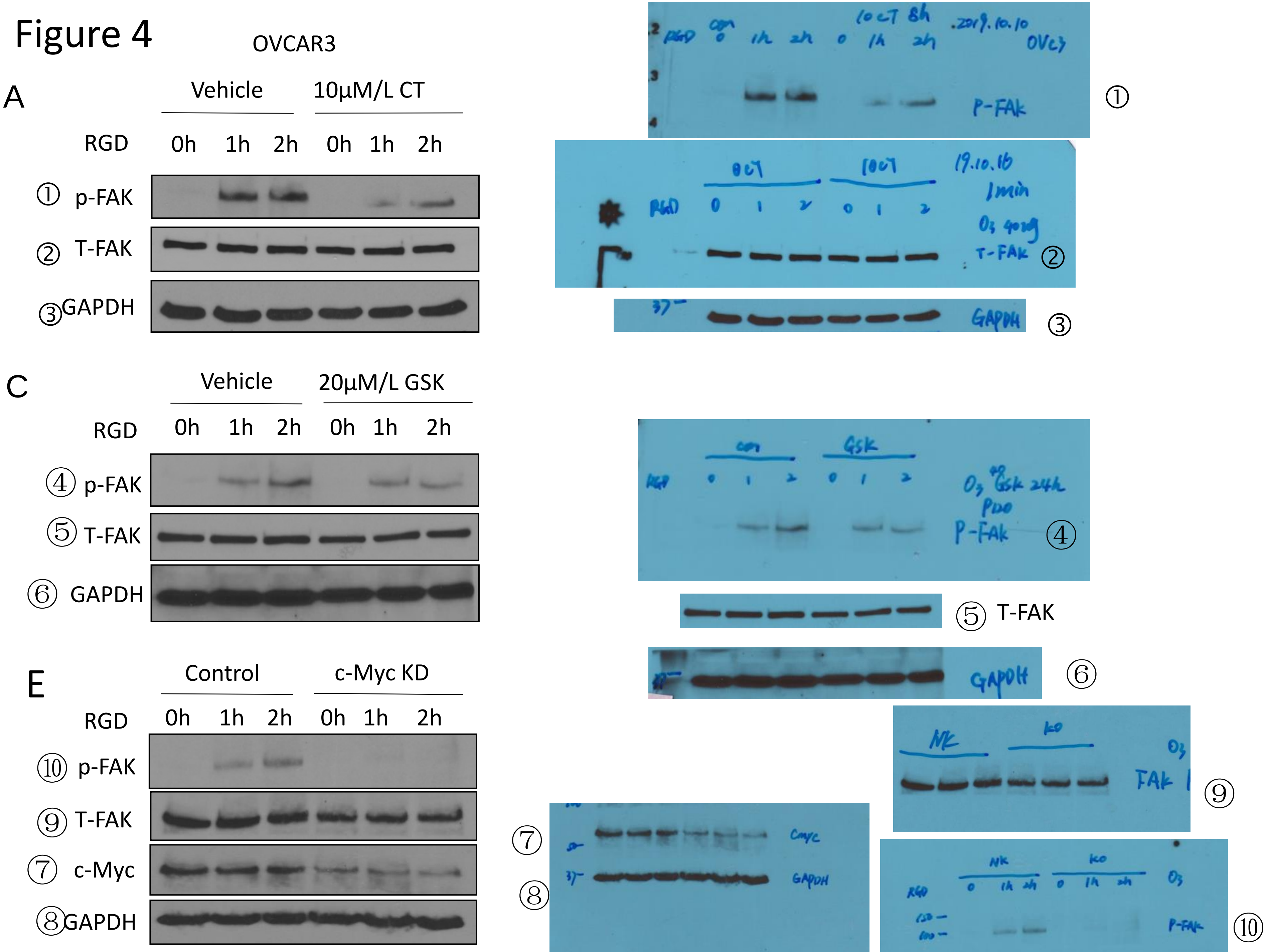
OVCAR3
A
Vehicle
10μM/L CT
RGD
0h 1h 2h 0h 1h 2h
① p-FAK
② T-FAK
③GAPDH
C
Vehicle
20μM/L GSK
RGD
0h 1h 2h 0h 1h 2h
④ p-FAK
⑤ T-FAK
⑥ GAPDH
E
Control
c-Myc KD
RGD
0h 1h 2h 0h 1h 2h
⑩ p-FAK
⑨ T-FAK
⑦ c-Myc
⑧GAPDH
①
②
③
④
⑤ T-FAK
⑥
⑨
⑦
⑧
⑩

Figure 4

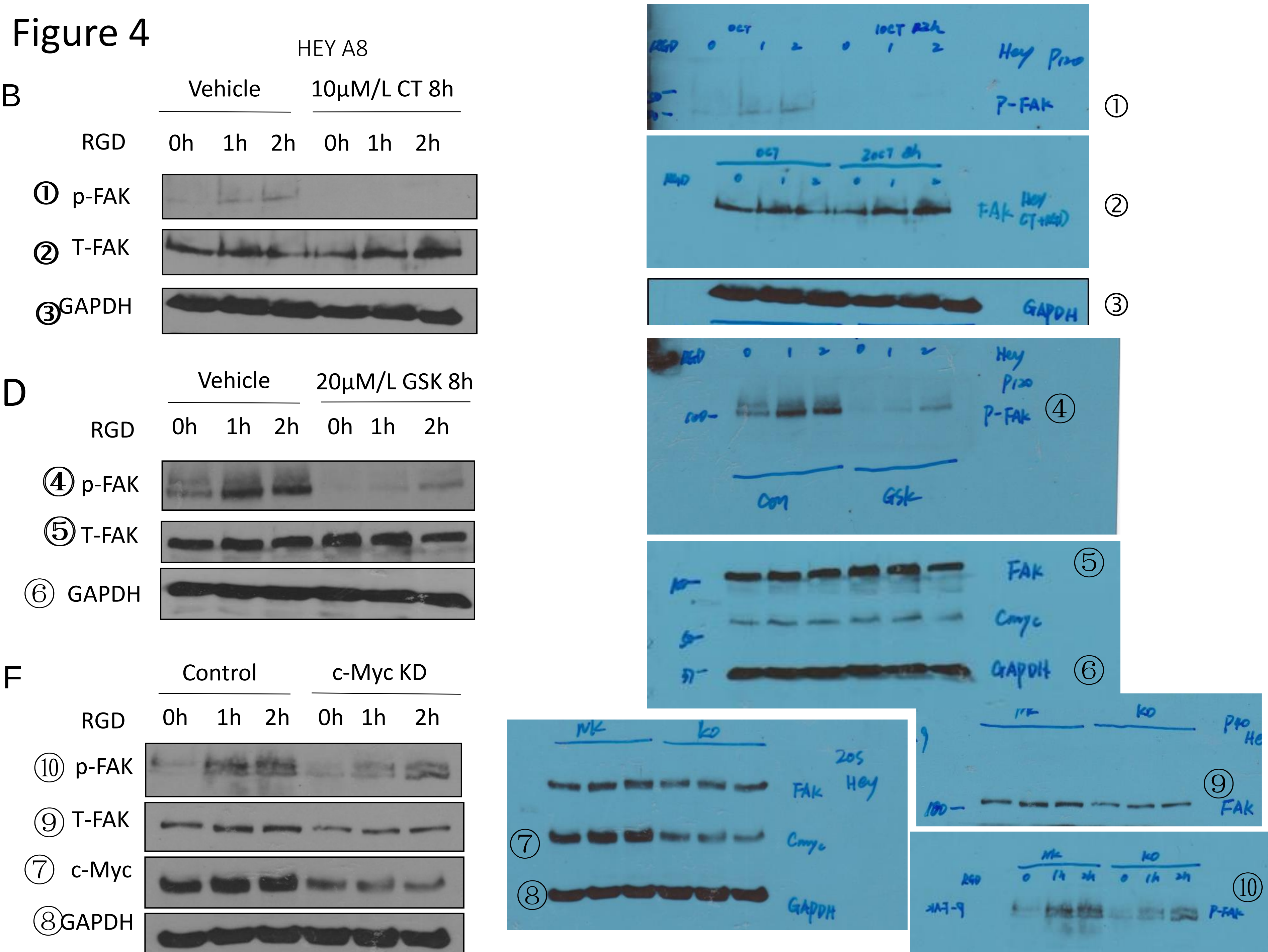
HEY A8
B
Vehicle
10μM/L CT 8h
RGD
0h 1h 2h 0h 1h 2h
① p-FAK
② T-FAK
③GAPDH
D
Vehicle
20μM/L GSK 8h
RGD
0h 1h 2h 0h 1h 2h
④ p-FAK
⑤ T-FAK
⑥ GAPDH
F
Control
c-Myc KD
RGD
0h 1h 2h 0h 1h 2h
⑩ p-FAK
⑨ T-FAK
⑦ c-Myc
⑧GAPDH
①
②
③
④
⑤
⑥
⑦
⑧
⑨
⑩

Figure 5

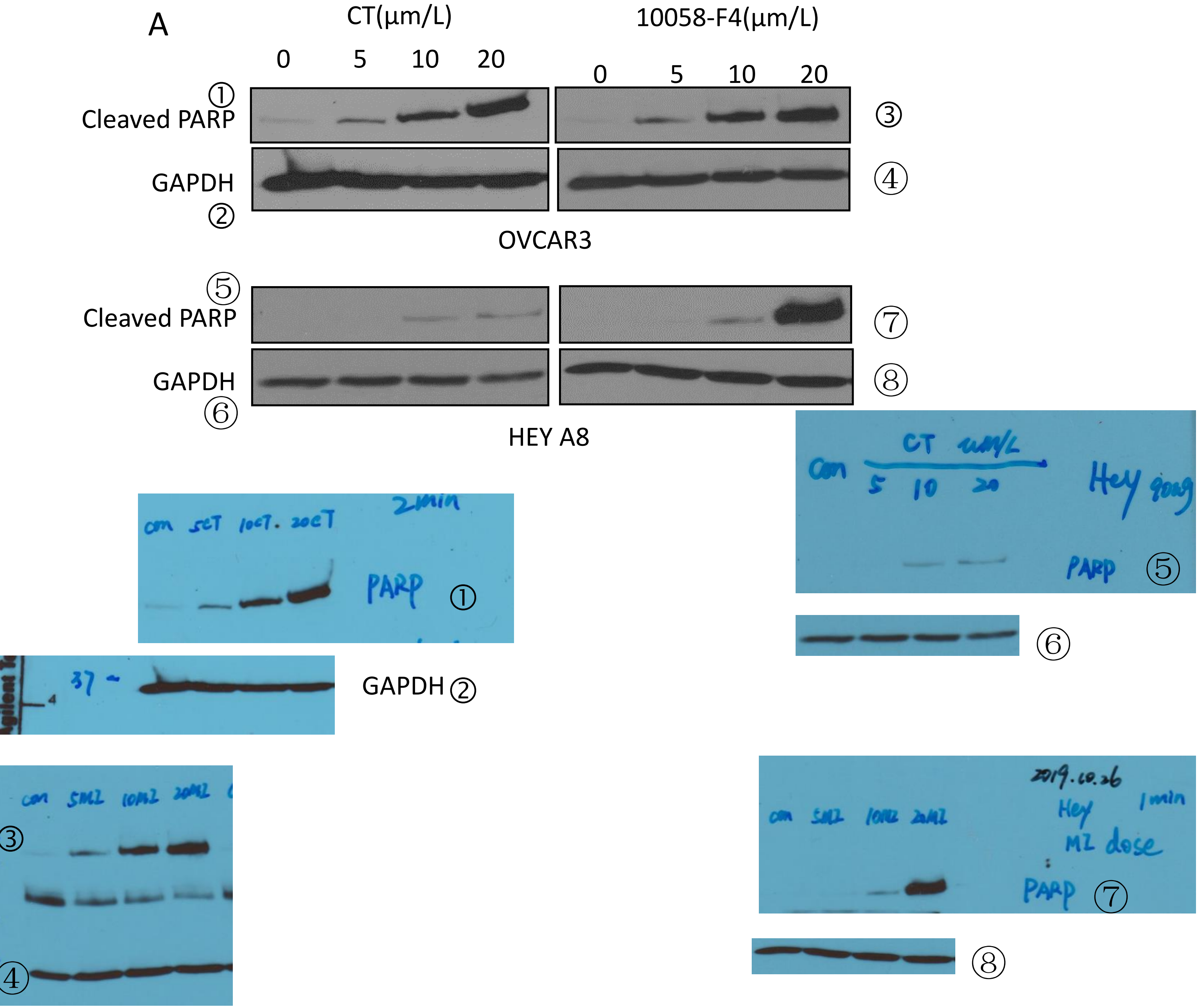

A
CT(μm/L)
0 5 10 20
10058-F4(μm/L)
0 5 10 20
① Cleaved PARP
GAPDH ②
③
④
OVCAR3
⑤ Cleaved PARP
GAPDH ⑥
⑦
⑧
HEY A8
PARP ①
GAPDH ②
③
④
PARP ⑤
⑥
PARP ⑦
⑧

Figure 5

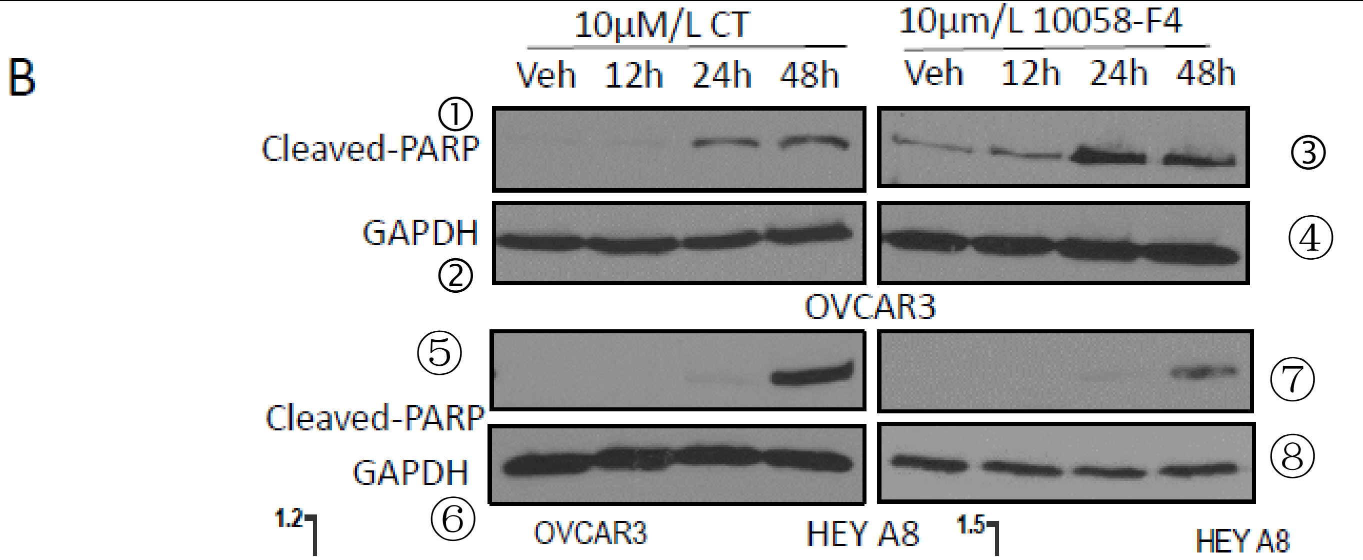
B
10µM/L CT
10µm/L 10058-F4
Veh 12h 24h 48h
Veh 12h 24h 48h
Cleaved-PARP
GAPDH
OVCAR3
Cleaved-PARP
GAPDH
OVCAR3
HEY A8
HEY A8
1.2
1.5
①
②
③
④
⑤
⑥
⑦
⑧

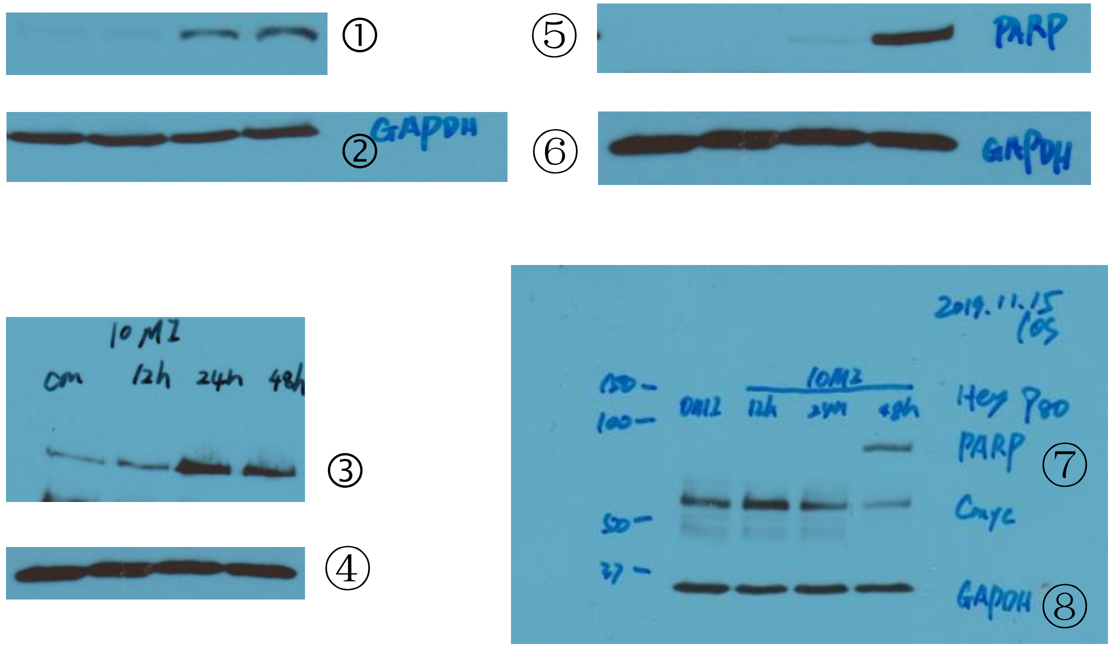
①
②
GAPDH
⑤
PARP
⑥
GAPDH
12h 24h 48h
③
④
2019.11.15
12h 24h 48h
PARP
⑦
Cmyc
GAPDH
⑧

Figure 5

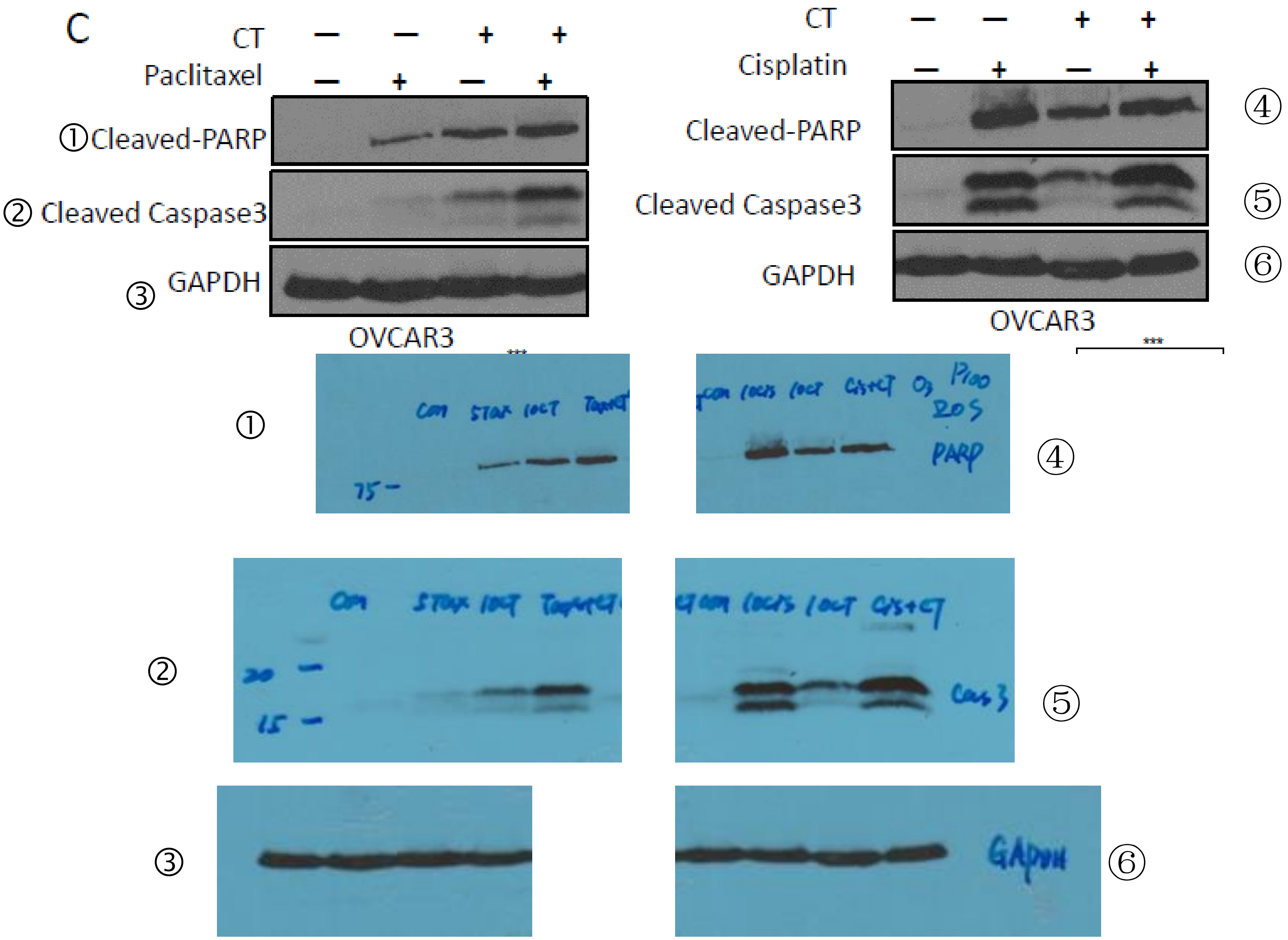
C
CT
Paclitaxel
①Cleaved-PARP
② Cleaved Caspase3
③ GAPDH
OVCAR3
CT
Cisplatin
Cleaved-PARP
Cleaved Caspase3
GAPDH
OVCAR3
④
⑤
⑥
①
④
②
⑤
③
⑥
PARP
GAPDH

Figure 5

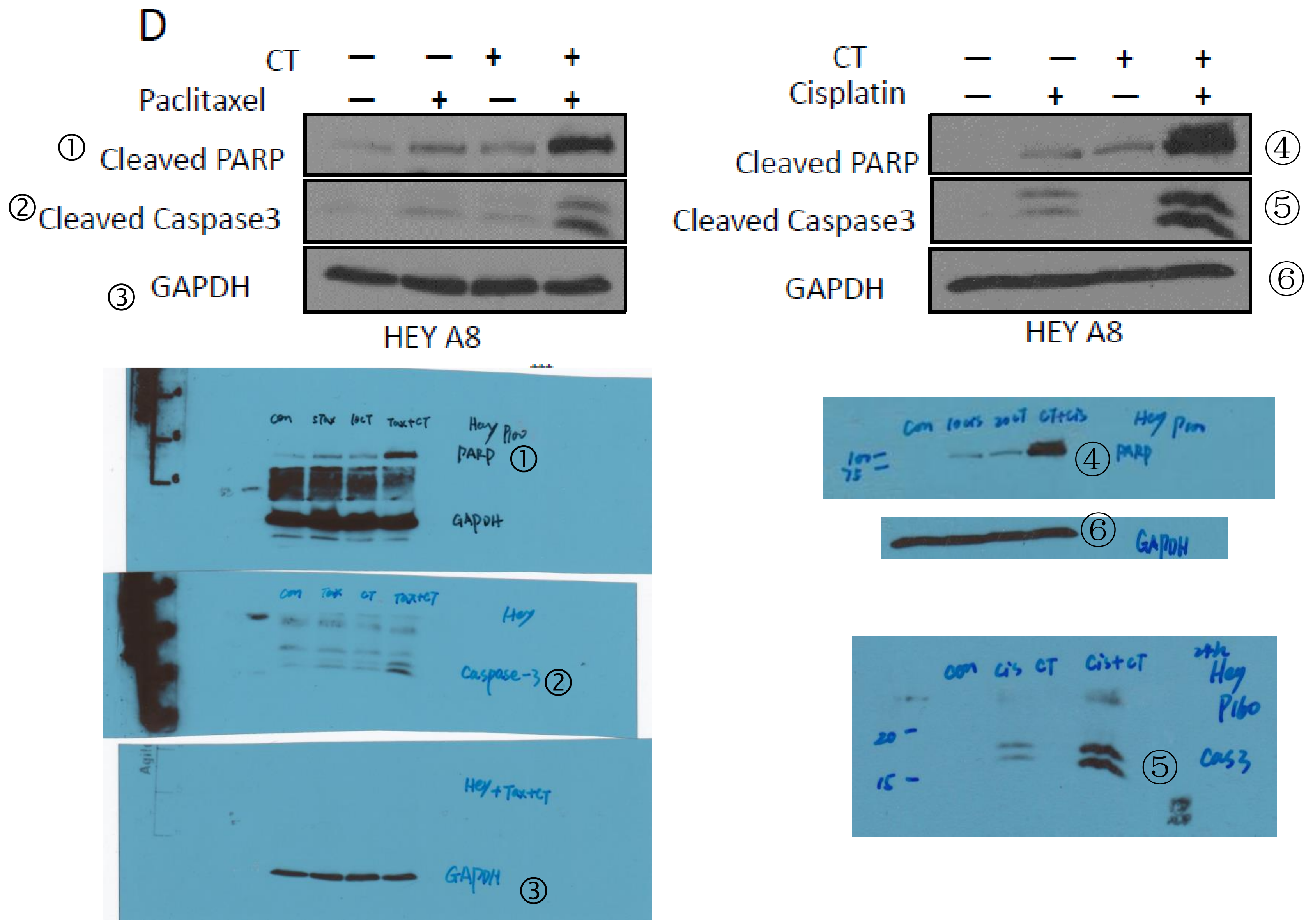

D
CT
Paclitaxel
① Cleaved PARP
② Cleaved Caspase3
③ GAPDH
HEY A8
CT
Cisplatin
Cleaved PARP ④
Cleaved Caspase3 ⑤
GAPDH ⑥
HEY A8
PARP ①
GAPDH
Caspase-3 ②
GAPDH ③
④ PARP
⑥ GAPDH
⑤

Figure 6

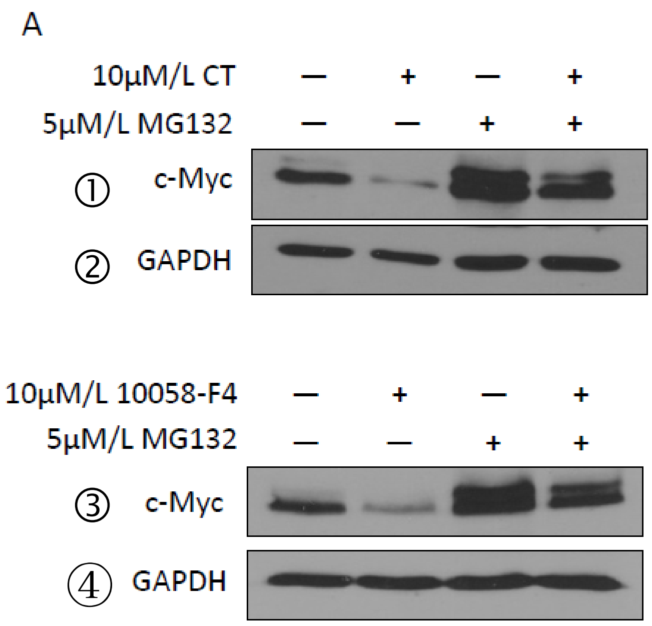

A
10μM/L CT — + — +
5μM/L MG132 — — + +
① c-Myc
② GAPDH
10μM/L 10058-F4 — + — +
5μM/L MG132 — — + +
③ c-Myc
④ GAPDH

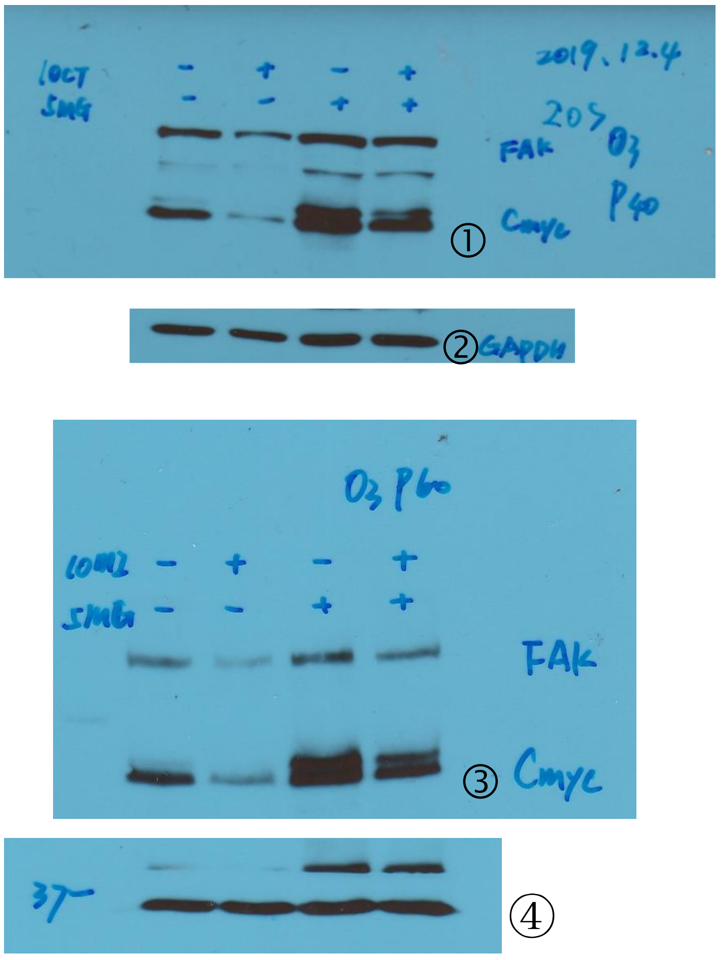

2019.12.4
FAK
Cmyc
①
②
GAPDH
O3 P60
FAK
③
Cmyc
④

Figure 6

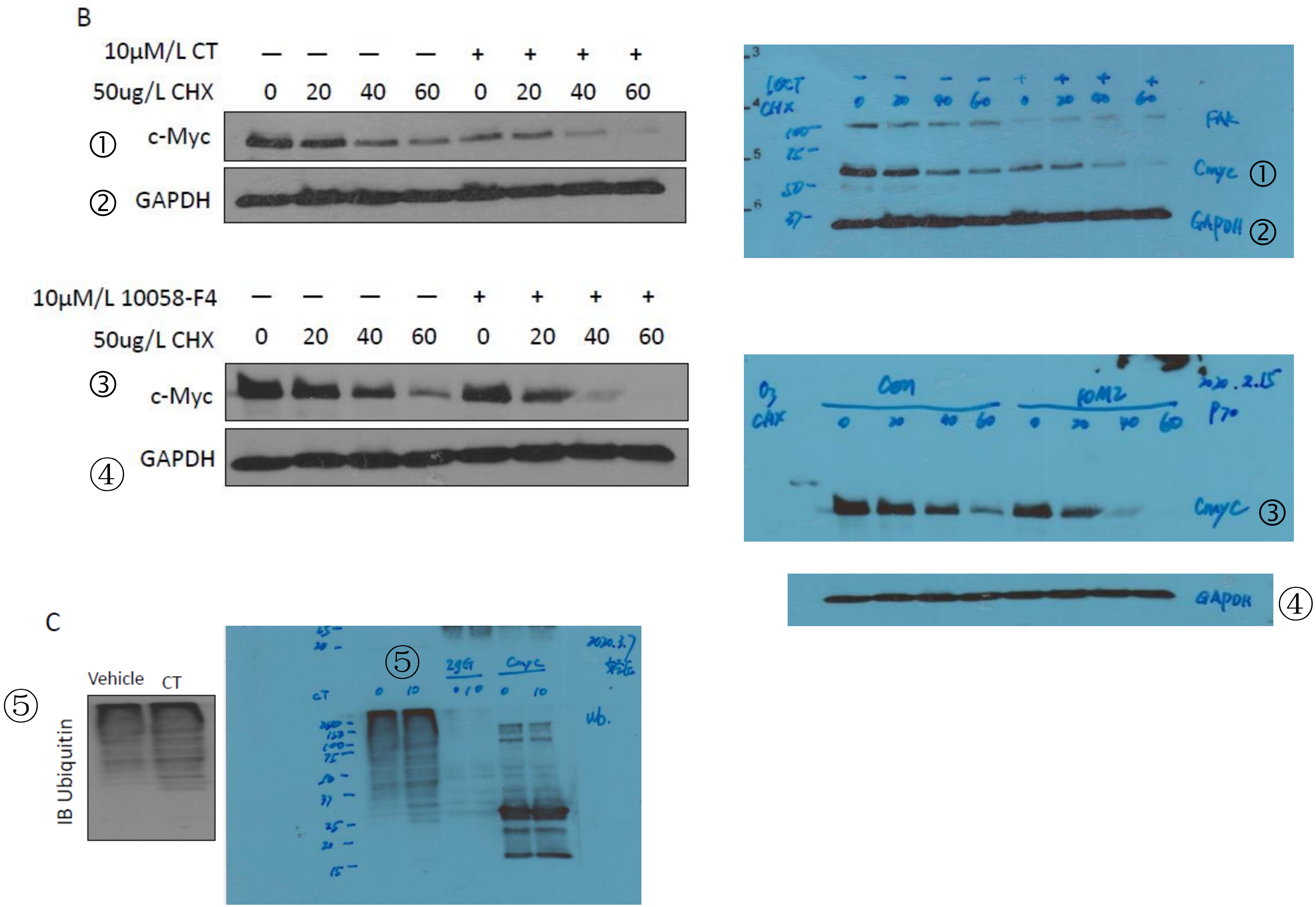

B
10μM/L CT — — — — + + + +
50ug/L CHX 0 20 40 60 0 20 40 60
① c-Myc
② GAPDH
10μM/L 10058-F4 — — — — + + + +
50ug/L CHX 0 20 40 60 0 20 40 60
③ c-Myc
④ GAPDH
FAK
Cmyc ①
GAPDH ②
Con
Cmyc ③
GAPDH ④
C
⑤
Vehicle
CT
IB Ubiquitin
IgG
Cmyc
ub.

Figure 6

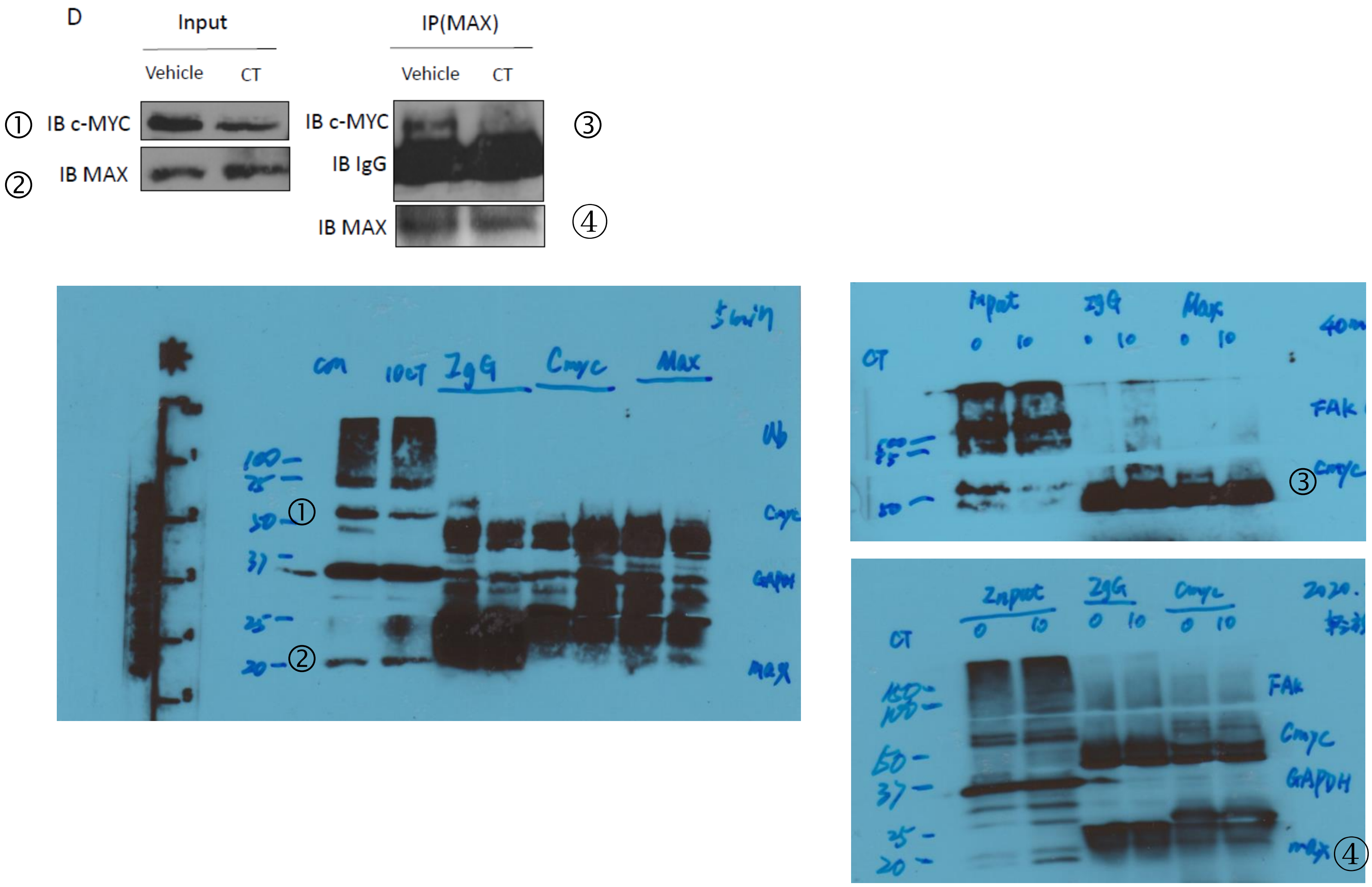
D
Input
IP(MAX)
Vehicle
CT
Vehicle
CT
① IB c-MYC
② IB MAX
IB c-MYC
IB IgG
IB MAX
③
④
①
②
③
④
FAK
GAPDH

Figure 6

C

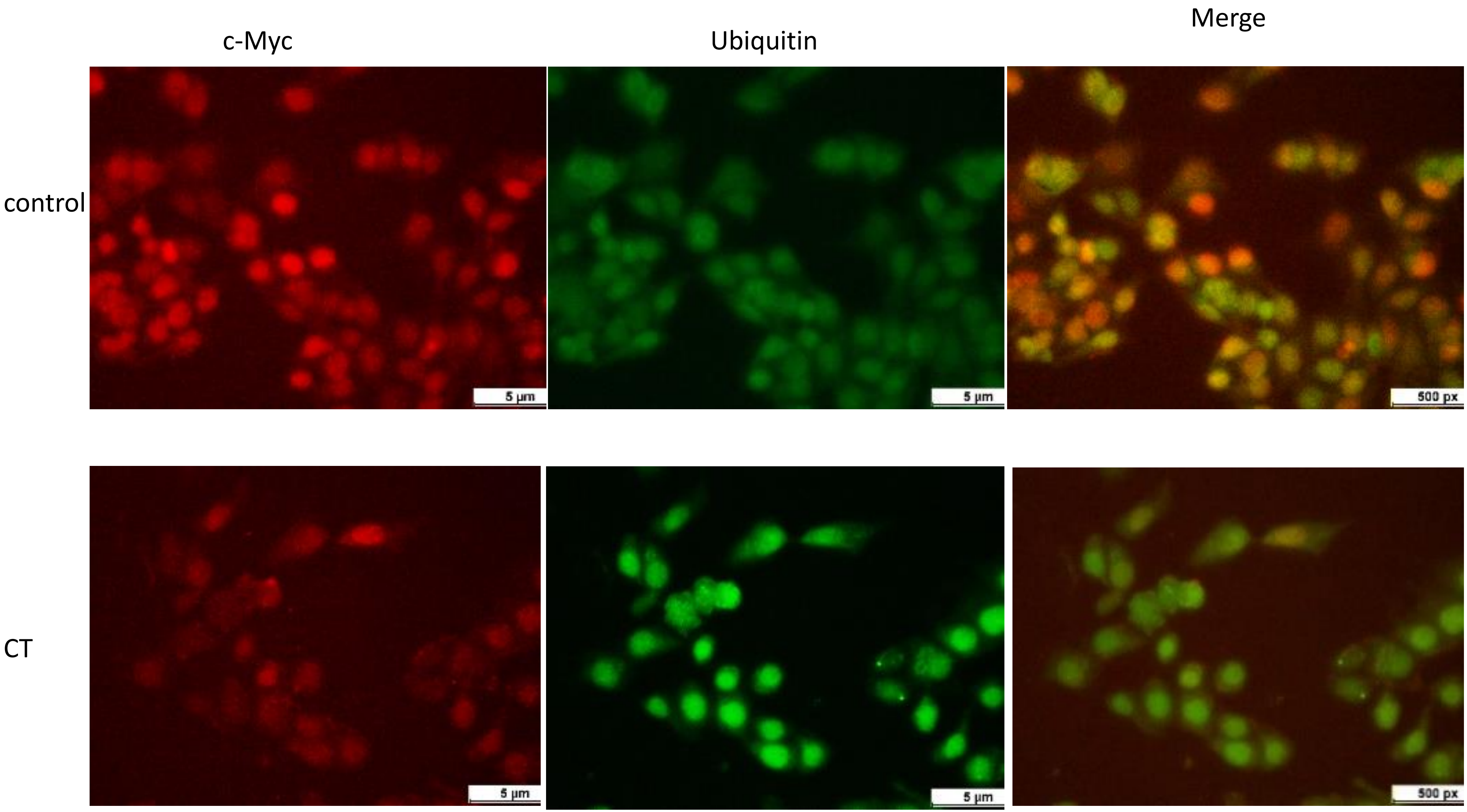

Figure 8

Control

CT

A

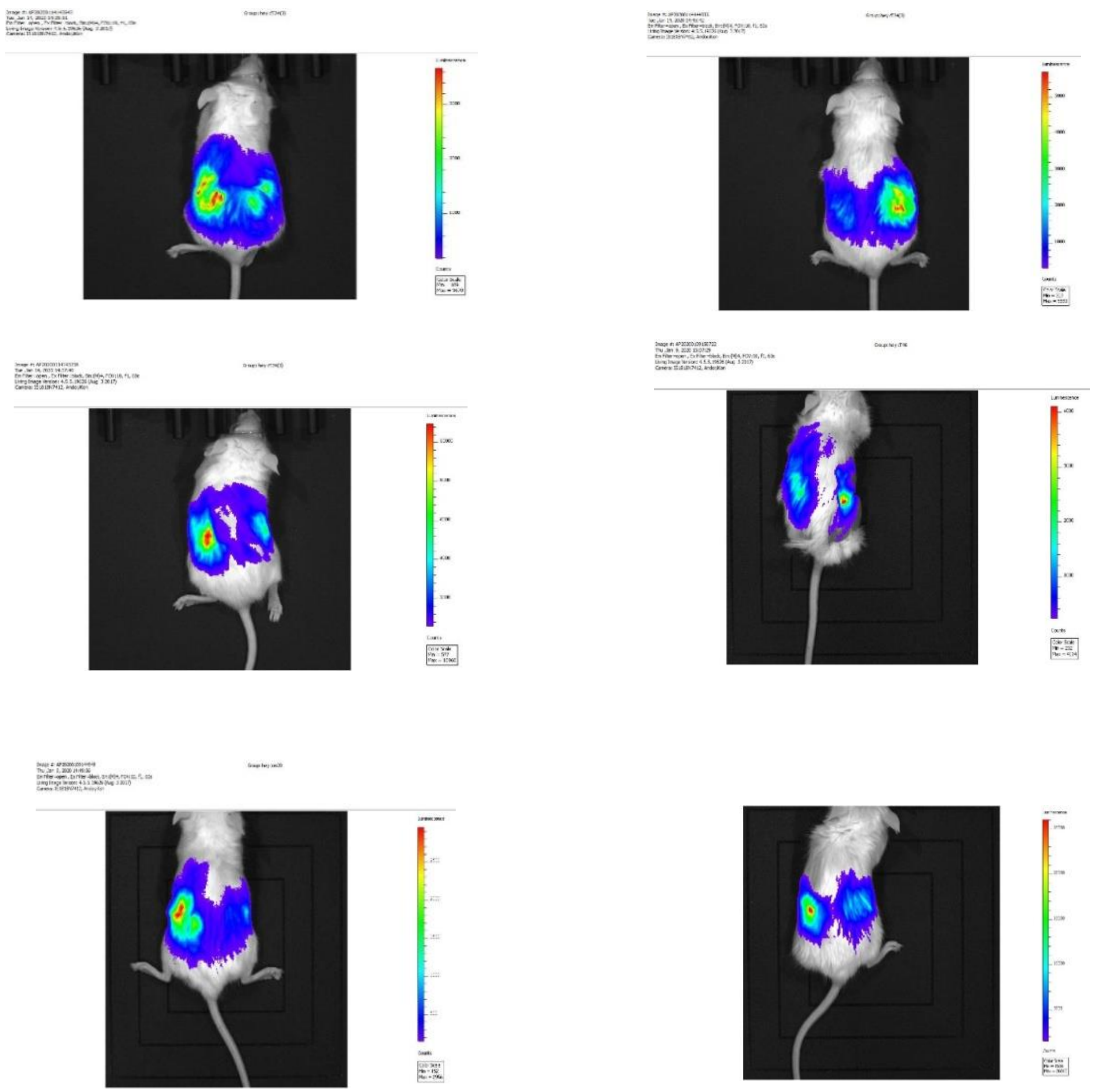

Figure 7

B

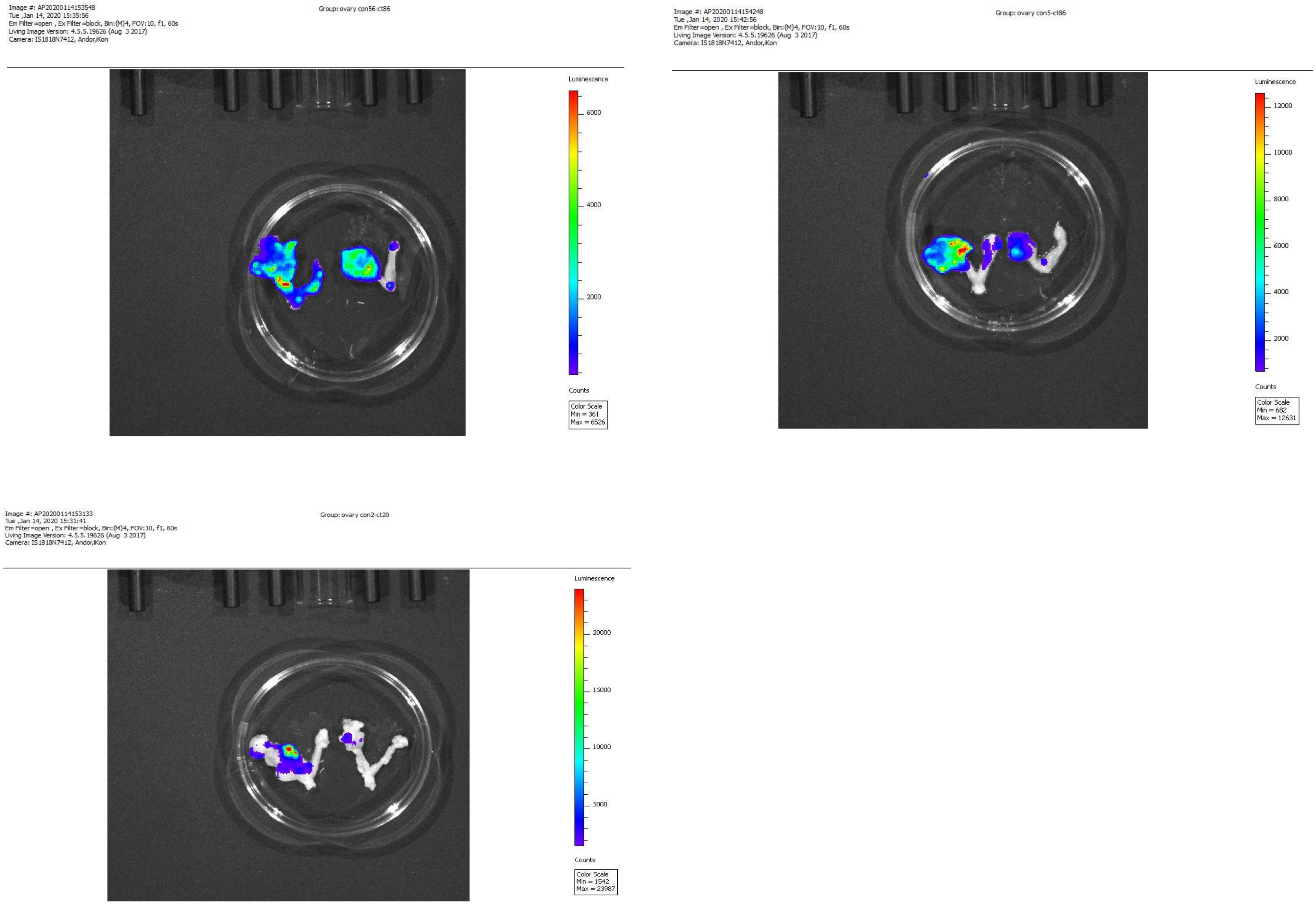

Image #: AP20200114153548
Tue ,Jan 14, 2020 15:35:56
Em Filter=open , Ex Filter=block, Bin:(M)4, FOV:10, f1, 60s
Living Image Version: 4.5.5.19626 (Aug 3 2017)
Camera: IS1818N7412, Andor,iKon
Group: ovary con56-ct86
Luminescence
6000
4000
2000
Counts
Color Scale
Min = 361
Max = 6526
Image #: AP20200114154248
Tue ,Jan 14, 2020 15:42:56
Em Filter=open , Ex Filter=block, Bin:(M)4, FOV:10, f1, 60s
Living Image Version: 4.5.5.19626 (Aug 3 2017)
Camera: IS1818N7412, Andor,iKon
Group: ovary con5-ct86
Luminescence
12000
10000
8000
6000
4000
2000
Counts
Color Scale
Min = 682
Max = 12631
Image #: AP20200114153133
Tue ,Jan 14, 2020 15:31:41
Em Filter=open , Ex Filter=block, Bin:(M)4, FOV:10, f1, 60s
Living Image Version: 4.5.5.19626 (Aug 3 2017)
Camera: IS1818N7412, Andor,iKon
Group: ovary con2-ct20
Luminescence
20000
15000
10000
5000
Counts
Color Scale
Min = 1542
Max = 23987

Figure 7

C

control CT

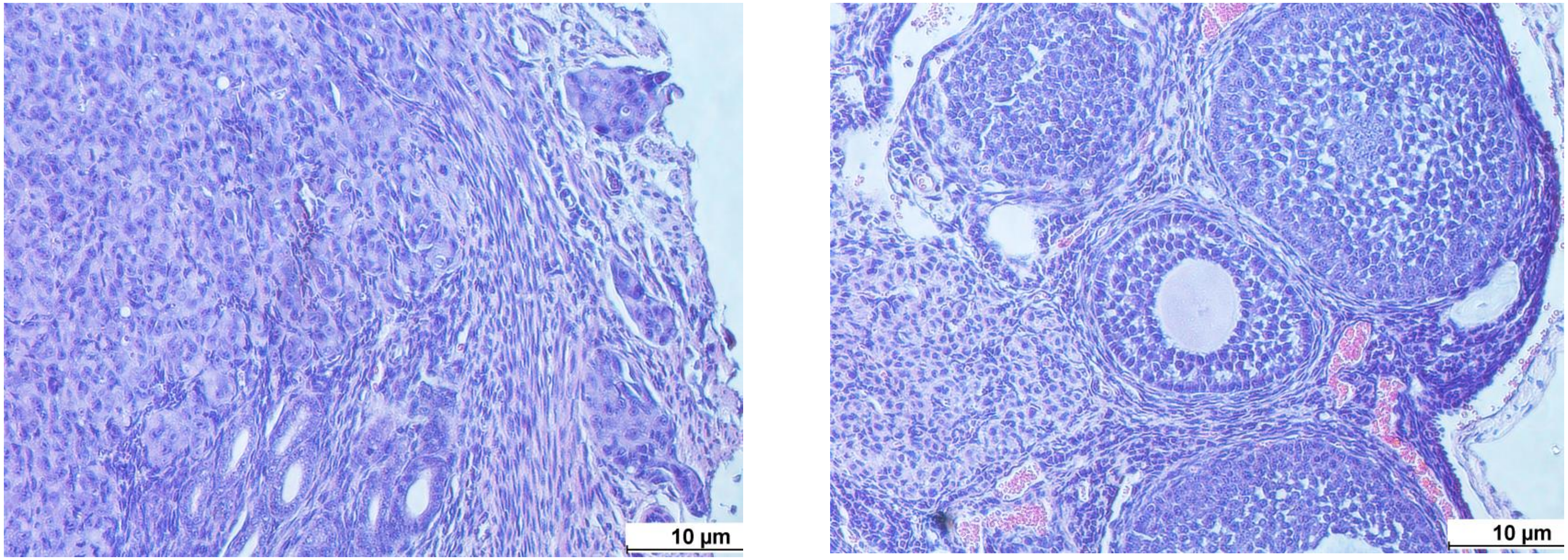

OVARIAN

Figure 7

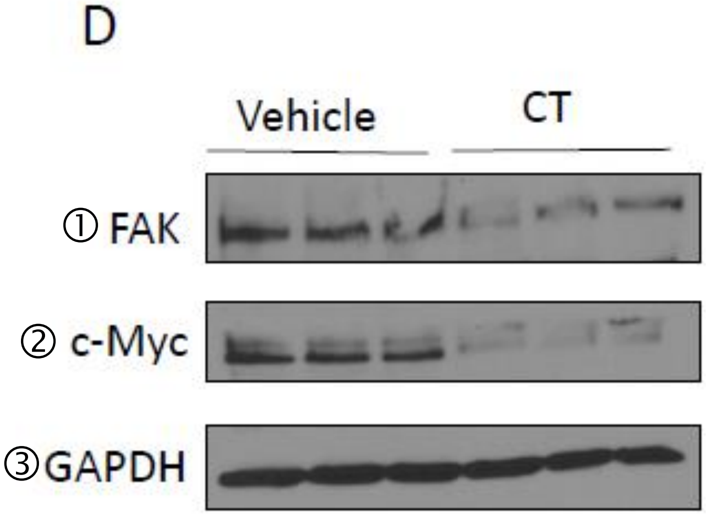
D
Vehicle
CT
① FAK
② c-Myc
③GAPDH

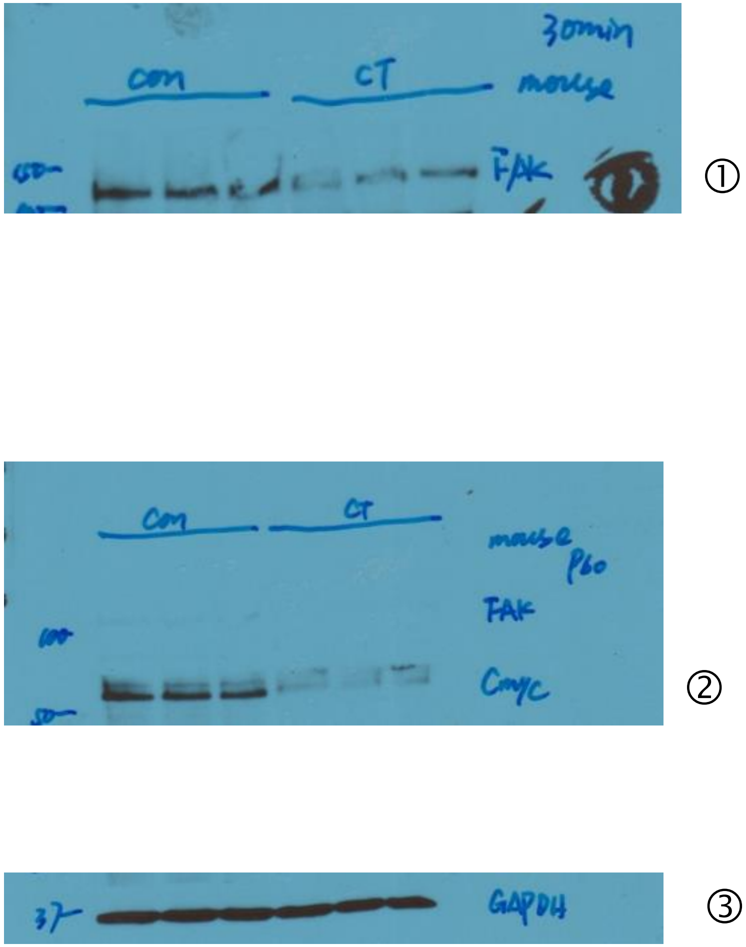
30min
con
CT
mouse
FAK
①
Con
CT
mouse
FAK
Cmyc
②
37
GAPDH
③

Figure 7

E

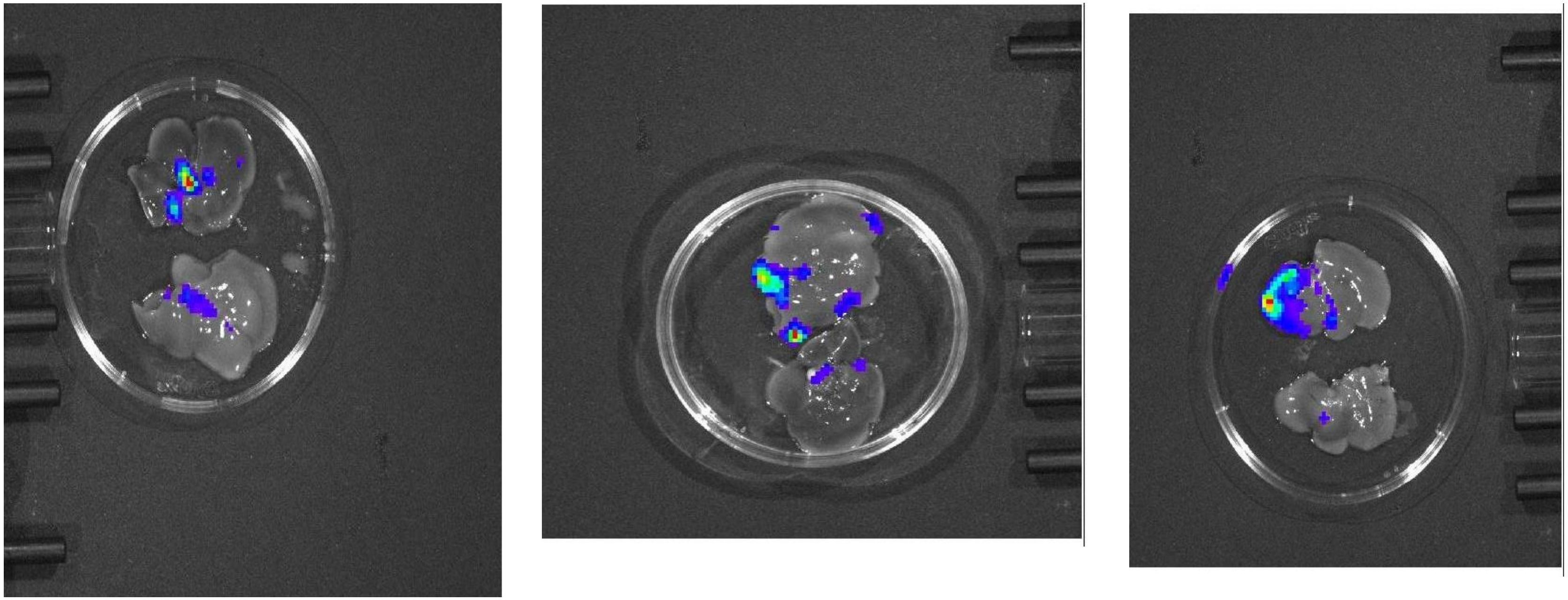

Figure 7

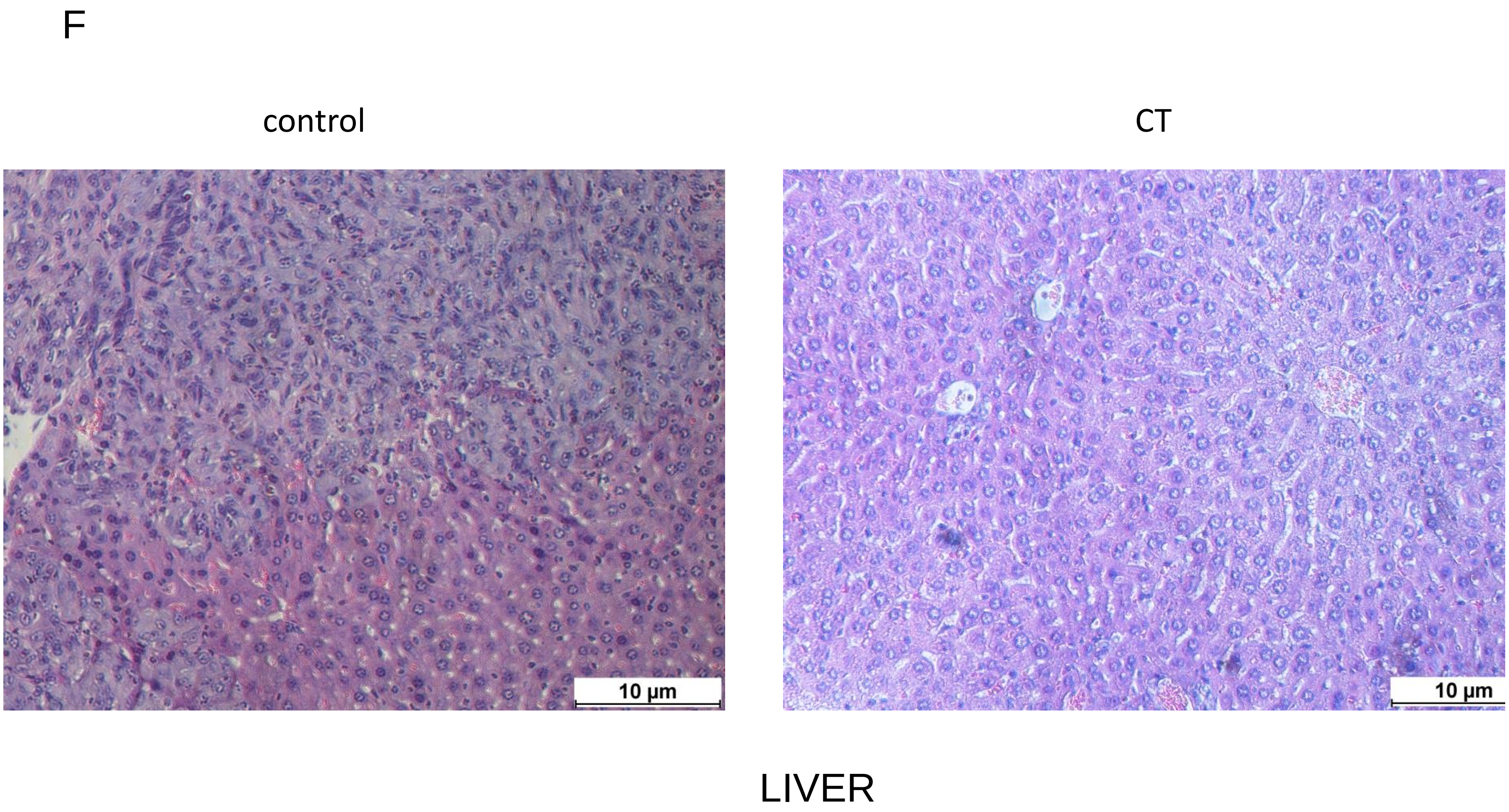

Figure S1

A

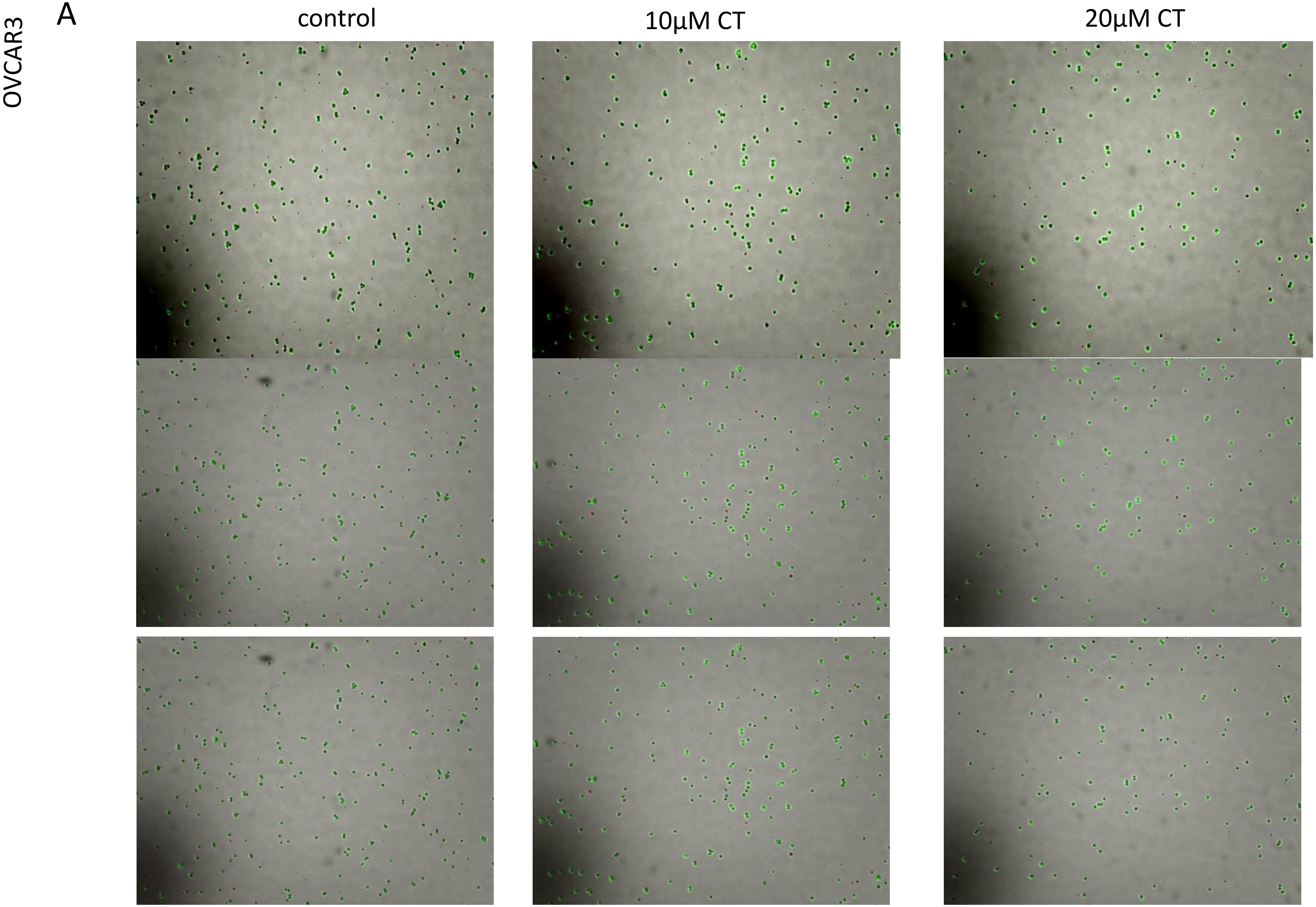

Figure S1

B

control | 10μM CT | 20μM CT

HEYA8

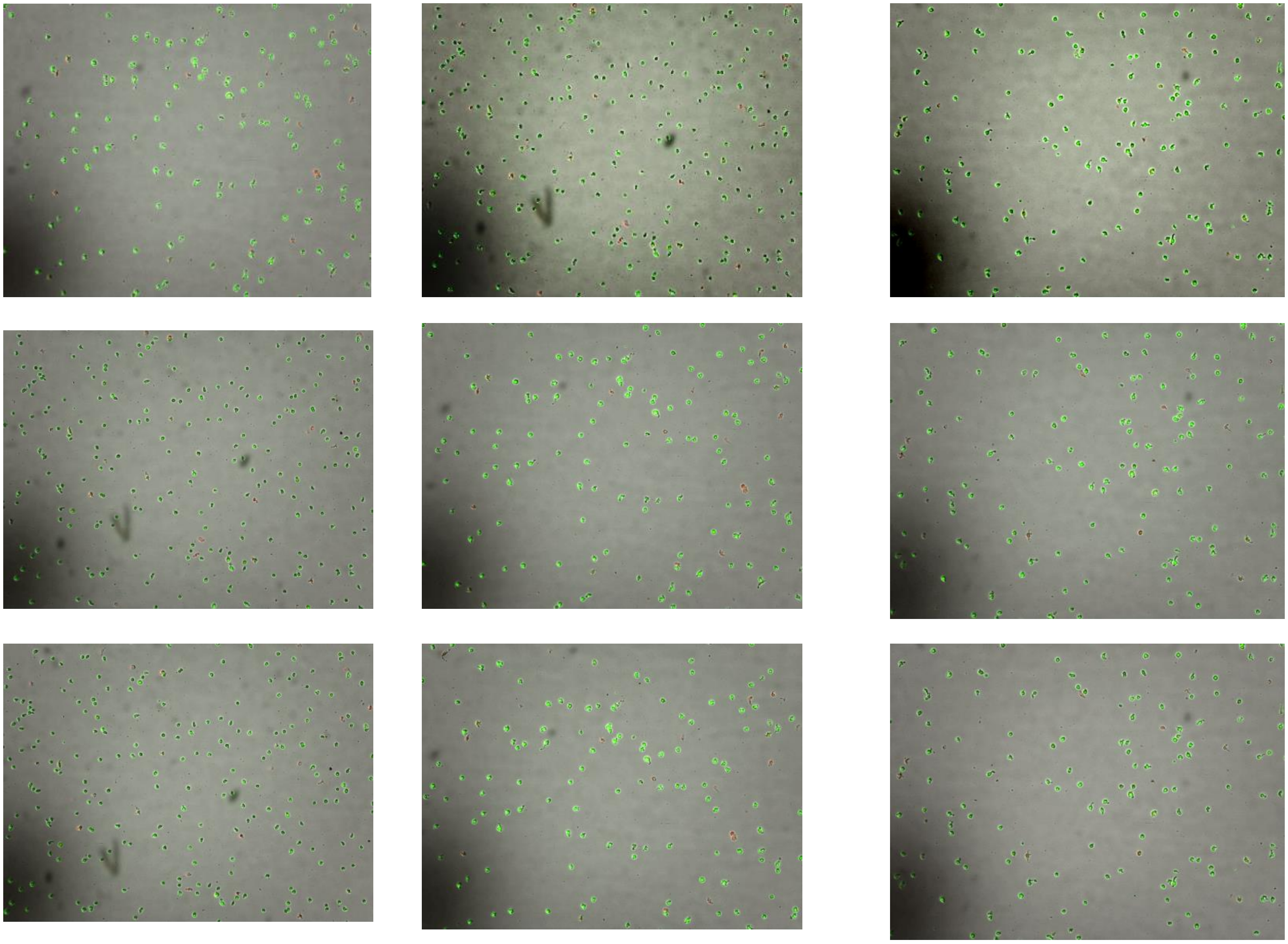

Figure S2

A

Control | Paclitaxel | CT | Paclitaxel + CT

Cleaved Caspase3

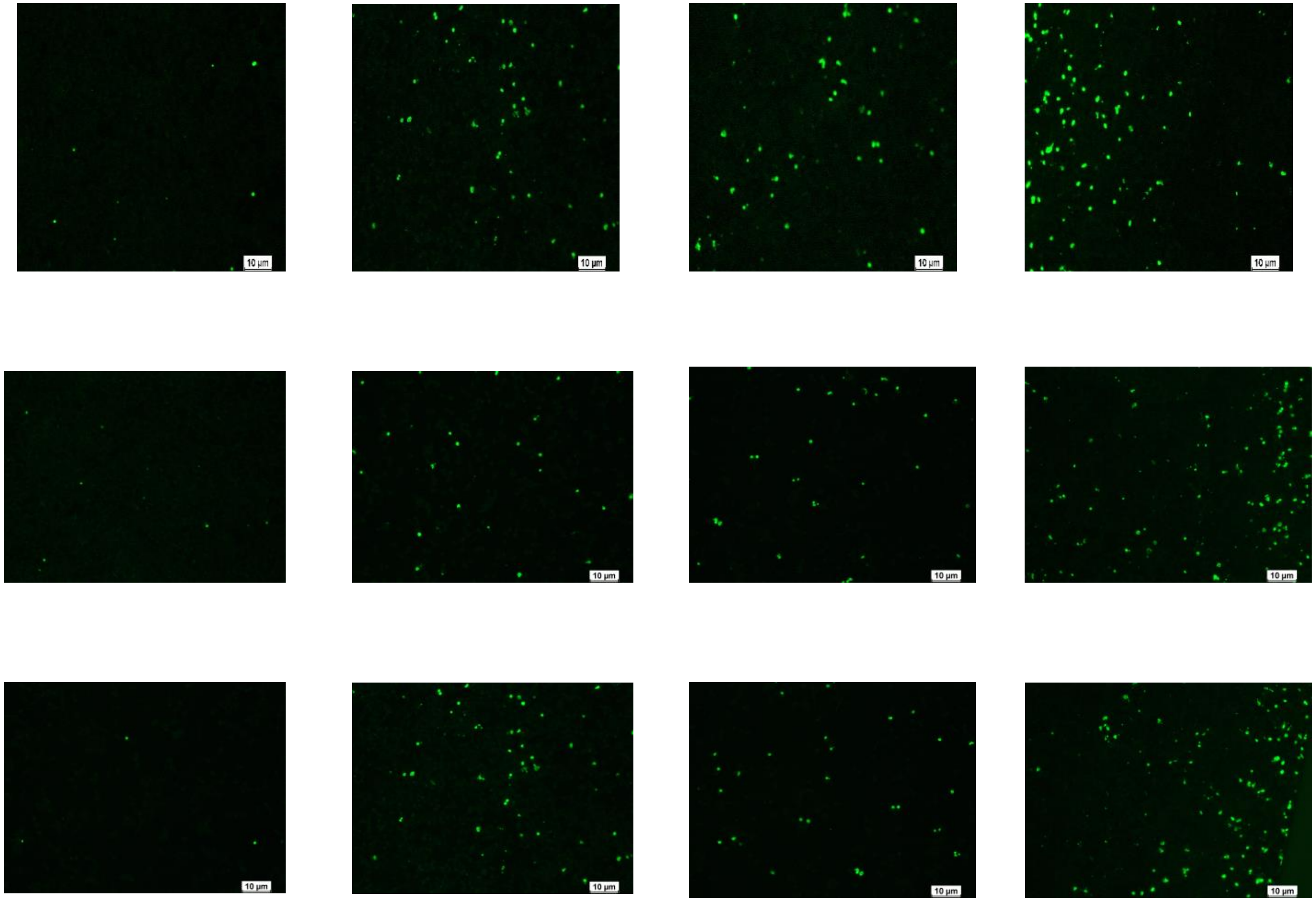

Figure S2

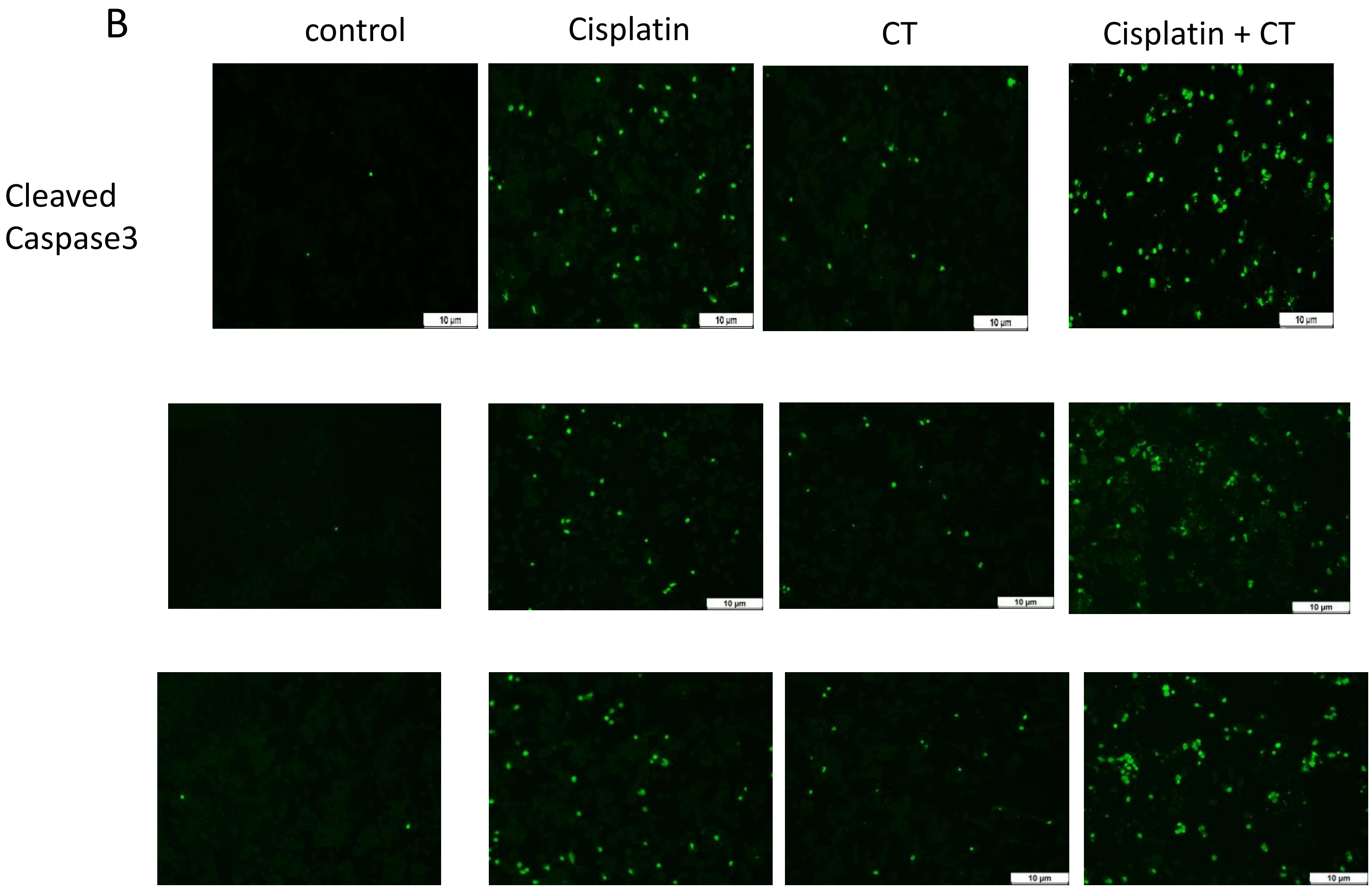

Supplement: Supplementary file 1 [file DataSheet2.pdf]
