# Supplementary material for: Cryptotanshinone inhibits ovarian tumor growth and metastasis by degrading c-Myc and attenuating the FAK signaling pathway
Source: Front Cell Dev Biol. 2022 Sep 28;10:959518. doi: 10.3389/fcell.2022.959518 (PMC9554091; doi:10.3389/fcell.2022.959518)
Supplement: Supplementary file 3 [file DataSheet1.PDF]

Cryptotanshinone inhibits ovarian tumor growth and metastasis by degrading c-Myc and attenuating the FAK pathway

Huijun Guo, Wenjing Zhang, Jiaxing Wang, Guannan Zhao, Yaohong Wang, Bingmei Zhu, Peixin Dong, Hidemichi Watari, Baojin Wang, Wei Li, Gabor Tigyi, Junming Yue

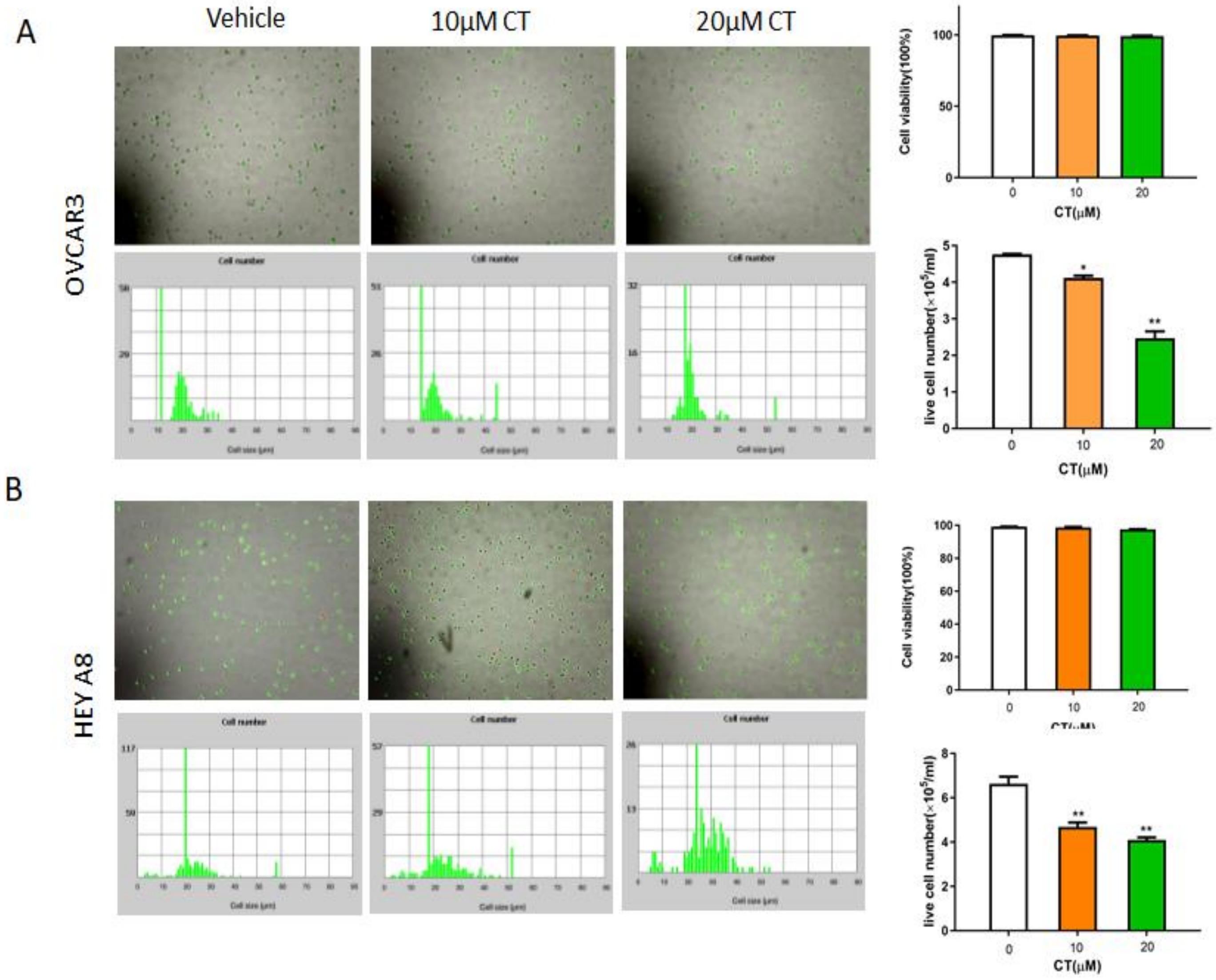

Figure S1. CT did not display cytotoxicity in ovarian cancer cells

(A) (B) Cell viability was detected by staining OVCAR3 (A) and HEY A8 (B) cells using acridine orange/propidium following different doses of CT treatment for 24 h. Live cells were stained in green and dead cells were stained in red. **$p<0.01$.

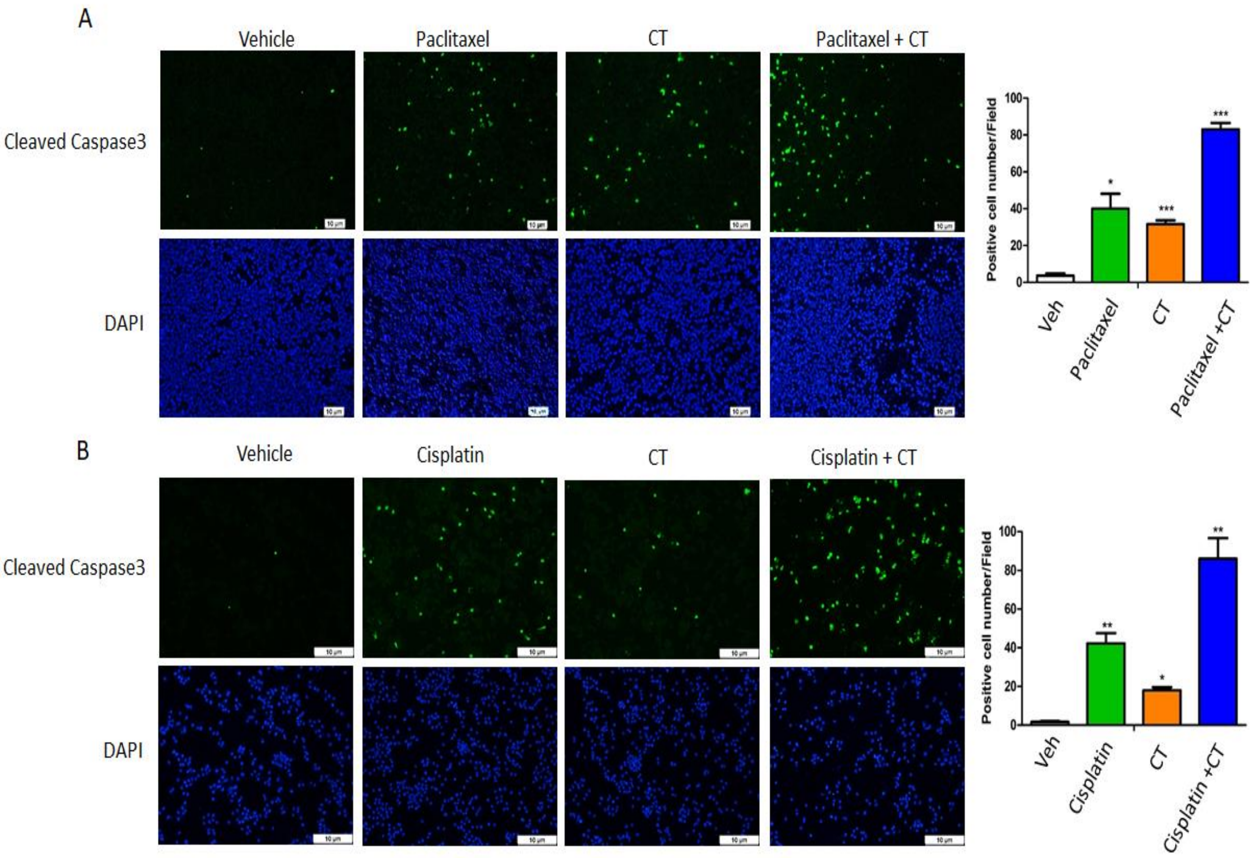

Figure S2. CT induced apoptosis in ovarian cancer cells.

(A) (B) Representative immunofluorescent images and quantification of apoptotic cells in OVCAR3 cells after 24 h treatment of CT with or without paclitaxel or cisplatin. Green signals indicated apoptotic cells. Scale bar, 10 μm. Error bars represent mean ± SD (n = 3, *p<0.05, **p<0.01, ***p < 0.001).

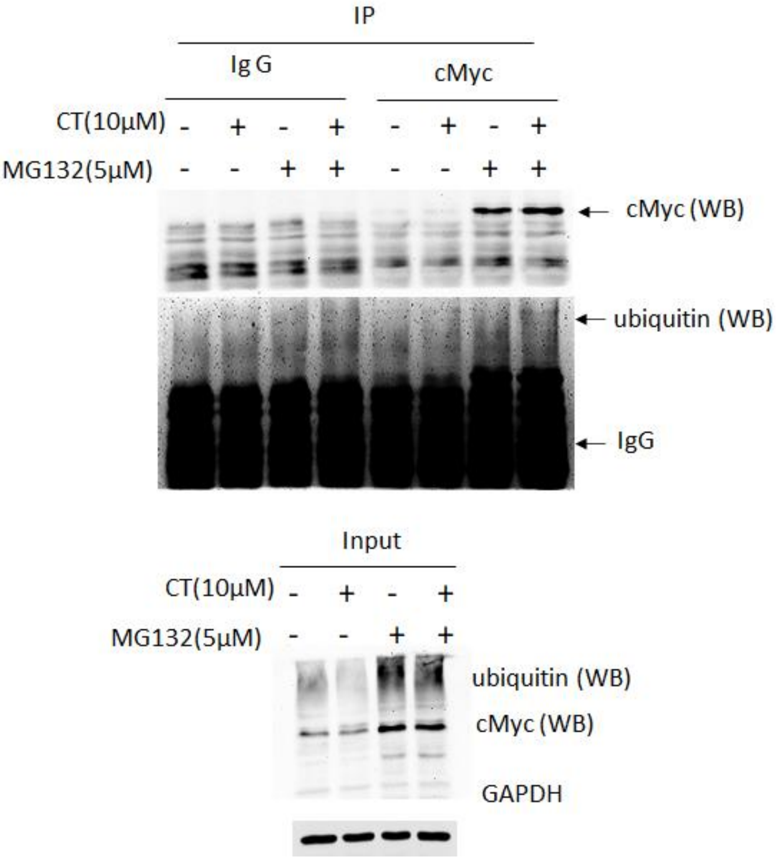

Figure S3. cMyc ubiquitination was detected by IP. OVCAR3 cells were treated with 10μM CT and 5μM MG132 and vehicle for 6h and then performed IP using cMyc antibody. The protein complex was detected by western blot (WB) using ubiquitin antibody.

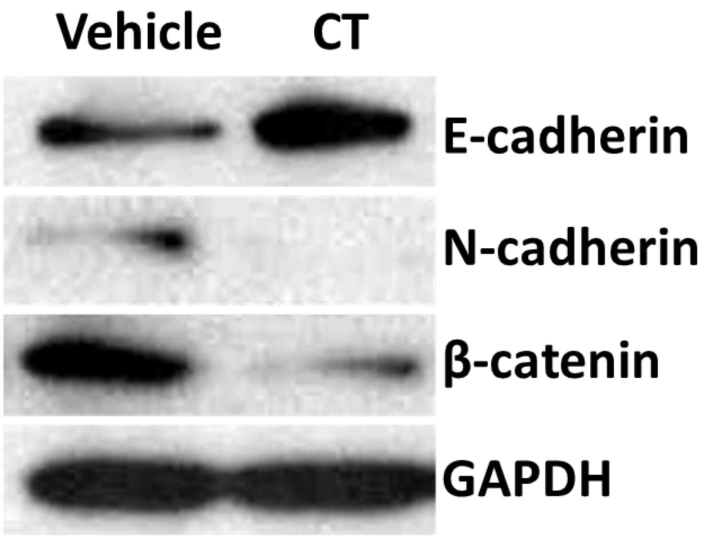

Figure S4. CT affects EMT markers in OC cells. OVCAR3 cells were treated with 10μM CT and vehicle for 24h and then the EMT marker proteins were detected by immunoblot.
